# Real-life ankle submovements and computer mouse use reflect patient-reported function in adult ataxias

**DOI:** 10.1093/braincomms/fcad064

**Published:** 2023-03-13

**Authors:** Nicole M Eklund, Jessey Ouillon, Vineet Pandey, Christopher D Stephen, Jeremy D Schmahmann, Jeremy Edgerton, Krzysztof Z Gajos, Anoopum S Gupta

**Affiliations:** Department of Neurology, Massachusetts General Hospital, Harvard Medical School, Boston, MA 02114, USA; Department of Neurology, Massachusetts General Hospital, Harvard Medical School, Boston, MA 02114, USA; School of Engineering and Applied Sciences, Harvard University, Allston, MA 02138, USA; Department of Neurology, Massachusetts General Hospital, Harvard Medical School, Boston, MA 02114, USA; Ataxia Center, Department of Neurology, Massachusetts General Hospital, Boston, MA 02114, USA; Department of Neurology, Massachusetts General Hospital, Harvard Medical School, Boston, MA 02114, USA; Ataxia Center, Department of Neurology, Massachusetts General Hospital, Boston, MA 02114, USA; Biogen Digital Health, Cambridge, MA 02142, USA; School of Engineering and Applied Sciences, Harvard University, Allston, MA 02138, USA; Department of Neurology, Massachusetts General Hospital, Harvard Medical School, Boston, MA 02114, USA; Ataxia Center, Department of Neurology, Massachusetts General Hospital, Boston, MA 02114, USA

**Keywords:** spinocerebellar ataxia, multiple system atrophy, biomarkers, wearable devices, outcome measures

## Abstract

Novel disease-modifying therapies are being evaluated in spinocerebellar ataxias and multiple system atrophy. Clinician-performed disease rating scales are relatively insensitive for measuring disease change over time, resulting in large and long clinical trials. We tested the hypothesis that sensors worn continuously at home during natural behaviour and a web-based computer mouse task performed at home could produce interpretable, meaningful and reliable motor measures for potential use in clinical trials. Thirty-four individuals with degenerative ataxias (spinocerebellar ataxia types 1, 2, 3 and 6 and multiple system atrophy of the cerebellar type) and eight age-matched controls completed the cross-sectional study. Participants wore an ankle and wrist sensor continuously at home for 1 week and completed the Hevelius computer mouse task eight times over 4 weeks. We examined properties of motor primitives called ‘submovements’ derived from the continuous wearable sensors and properties of computer mouse clicks and trajectories in relationship to patient-reported measures of function (Patient-Reported Outcome Measure of Ataxia) and ataxia rating scales (Scale for the Assessment and Rating of Ataxia and the Brief Ataxia Rating Scale). The test–retest reliability of digital measures and differences between ataxia and control participants were evaluated. Individuals with ataxia had smaller, slower and less powerful ankle submovements during natural behaviour at home. A composite measure based on ankle submovements strongly correlated with ataxia rating scale scores (Pearson’s *r* = 0.82–0.88), strongly correlated with self-reported function (*r* = 0.81), had high test–retest reliability (intraclass correlation coefficient = 0.95) and distinguished ataxia and control participants, including preataxic individuals (*n* = 4) from controls. A composite measure based on computer mouse movements and clicks strongly correlated with ataxia rating scale total (*r* = 0.86–0.88) and arm scores (*r* = 0.65–0.75), correlated well with self-reported function (*r* = 0.72–0.73) and had high test–retest reliability (intraclass correlation coefficient = 0.99). These data indicate that interpretable, meaningful and highly reliable motor measures can be obtained from continuous measurement of natural movement, particularly at the ankle location, and from computer mouse movements during a simple point-and-click task performed at home. This study supports the use of these two inexpensive and easy-to-use technologies in longitudinal natural history studies in spinocerebellar ataxias and multiple system atrophy of the cerebellar type and shows promise as potential motor outcome measures in interventional trials.

## Teaser

Eklund *et al*. describe a new approach for capturing motor impairment in ataxia, using an ankle sensor worn continuously at home. Submovement measures were highly reliable, strongly related to patient function and differed in preataxic individuals and controls, demonstrating potential as a sensitive motor outcome measure.

## Graphical Abstract

**Graphical Abstract:**
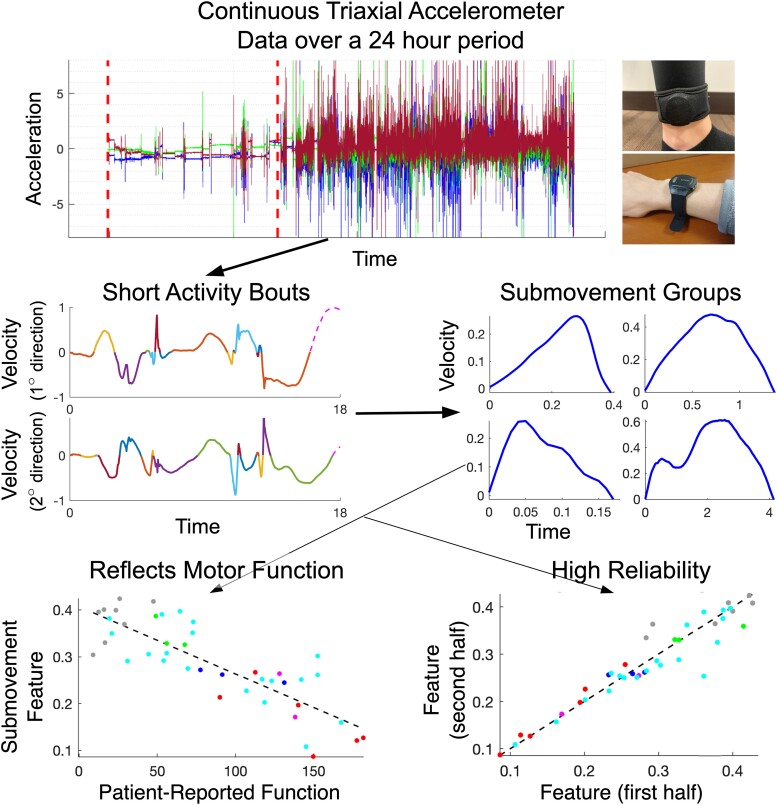

## Introduction

Novel therapeutic modalities are now aimed at proximal disease mechanisms in degenerative ataxias, for example targeting expression of genes containing disease-related triplet repeat expansions in spinocerebellar ataxias (SCAs)^1-3^ and alpha-synuclein aggregation in multiple system atrophy (MSA).^4^ One major barrier to the successful development of therapies that slow or stop progression of movement disorders is a lack of tools that can reliably quantify disease worsening over the duration of a clinical trial. Clinician-performed disease rating scales, which are composed of semiquantitatively scored neurological examination tasks, are subjective and coarse and capture the state of the individual at a snapshot in time.^5^ Thus, these rating scales have sources of variance that limit their sensitivity for measuring disease change, requiring long and large clinical trials to demonstrate efficacy.^6,7^ This raises particular challenges for trials in rare diseases. Novel quantitative assessment tools have the potential to objectively and more precisely measure disease severity. However, when measurements are collected infrequently, as is the case for in-person assessments, these tools cannot account for day-to-day and moment-to-moment variability in the disease state and have limited ability to account for variability in behavioural task performance and measurement error. Furthermore, it can be unclear whether the measured disease characteristics reflect aspects of behavioural change that are meaningful to patients.

Assessments that are conducted remotely in a participant’s home environment, using powerful yet inexpensive and familiar digital devices, are a promising approach.^8-14^ Behaviour can be sampled frequently and over multiple days, enabling measurements that can account for short-term variability to produce more reliable and precise estimates of disease severity.^15-18^ Assessment approaches that passively capture and analyze natural behaviour at home have access to different information than task-based approaches and may produce more ecologically valid and meaningful measures. At-home assessments using inexpensive and easy-to-use tools also have the potential to reduce cost and burden on participants and clinical teams, while increasing access to clinical research and clinical care for traditionally underserved populations.

It was recently shown that a wearable sensor-based technology that analyses natural wrist behaviour based on characteristics of motor primitives called ‘submovements’ (SMs) can sensitively measure changes in motor function over time in ataxia telangiectasia.^19^ It was also reported that analysis of computer mouse trajectories and clicks enabled accurate estimates of ataxia and parkinsonism severity and was able to sensitively detect disease change over time in individuals with ataxia, based on ‘in-clinic’ data collection.^20^ Here, we test the hypothesis that analysis of natural ankle and wrist movements as well as computer mouse movements at home in individuals with SCAs and MSA of the cerebellar type (MSA-C) can produce interpretable motor measures that reflect meaningful patient-reported function, have high reliability and are feasible for use in clinical trials.

## Materials and methods

### Recruitment and consent

The study protocol was approved by Partners Healthcare Research Committee Institutional Review Board (No. 2019P003458). Informed consent was obtained from all participants prior to participating in this research study according to the Declaration of Helsinki.

Participants with SCA (types 1, 2, 3 or 6) or MSA-C were recruited from the Massachusetts General Hospital (MGH) Ataxia Center and through the National Ataxia Foundation (NAF) website. Participants’ spouses were recruited as controls if they had no known risk factors for ataxia.

To participate, subjects were required to be able to walk without human assistance (canes, walkers, etc. were acceptable), move a computer mouse to click objects on a computer screen, be native English speakers and be at least 18 years old. Participants with other conditions that affected speech and motor function or would interfere with their ability to participate safely were excluded from the study.

Forty-three participants consented to participate in the research study between November 2019 and May 2022, and 42 were included in the analysis (four SCA1, two SCA2, 20 SCA3, three SCA6, six MSA-C and eight age-matched controls, Table 1). One participant was excluded from analysis for having misunderstood instructions, performing the computer mouse task for one session and wearing sensors for 1 day.

**Table 1:** Participant demographic and clinical information

Age group	Subject	Sex	Diagnosis	BARS total	SARA total	MDS-UPDRS total	PROM-Ataxia total	EQ-5D-VAS (100–0)
**30–40**	1	F	SCA3	0.75	0.8	1	73	67.5
	2	M	SCA3	5.75	5.5	6.5	64.5	82.5
	3	F	Control	0	0	0	23.5	100
	4	F	SCA3	19.75	19.9	41.5	107	83
	5	F	SCA3	14	15.8	38.5	123	77.5
	6	F	SCA3	0.25	1.3	3.5	54	73
**41–50**	7	F	SCA3	15	18.1	41	117	35
	8	M	SCA3	5	6.5	19.5	69.5	60
	9	M	SCA3	17	17.5	28	152.5	78.5
	10	M	SCA3	21	25.8	50.5	167.5	67.5
	11	F	Control	0	0	2	16	82.5
	12	M	SCA3	5.75	5.8	11.5	19.5	77.5
	13	F	Control	0	0	0	9	97
	14	F	SCA1	11	13.4	36.2	131	50
	15	F	SCA3	4.25	4.8	9	53	92.5
	16	F	SCA2	9.5	11.8	26	128	77.5
**51–60**	17	F	Control	0	0	0	12.5	94
	18	M	SCA2	17	16.8	29.5	138	89.5
	19	F	SCA3	13.75	16.3	22	118.5	44.5
	20	M	MSA-C	16.5	19.3	48	177.5	62
	21	M	Control	0	0	0	26	87.5
	22	F	SCA3	8.75	11.5	23.5	72.5	81
	23	M	SCA1	13.75	14.1	37	77.5	50
	24	F	MSA-C	18.5	18.7	31.7	181.5	57
	25	M	SCA3	10.5	9.8	15.5	31	87
	26	M	SCA3	10.75	9.8	18	21	94.5
	27	F	SCA6	5.5	5	12	49	90.5
	28	F	SCA3	9.25	13.5	21	56	93.5
	29	M	SCA3	1.5	1.5	7	44.5	88.5
**61–72**	30	M	SCA3	11.75	11.5	16.5	152.5	27.5
	31	M	MSA-C	17.75	20.2	56.5	149.5	38
	32	F	Control	0	0	0	47.5	81
	33	F	SCA1	9.75	11	17.5	91.5	50
	34	F	Control	0	0	1	29	86.5
	35	F	MSA-C	7.25	8.3	14.5	112.5	40
	36	F	MSA-C	13	16	25.5	140	71.5
	37	M	Control	1.5	3.5	7	16.5	90
	38	M	MSA-C	14.5	16	28.5	90	85
	39	M	SCA3	16.75	16.3	40	145	88.5
	40	M	SCA6	2.5	1	6	67.5	96.5
	41	M	SCA6	5.5	3	4	56	90
	42	F	SCA3	14.75	17	24	142	79

Average SARA/BARS scores from the two virtual assessors are shown. Some total scores contain fractions of a point due to the linear scaling procedure applied for missing subscores (see ‘Materials and methods’ section). BARS, Brief Ataxia Rating Scale; EQ-5D-VAS, EuroQOL-5D-Visual Analogue Scale; PROM-Ataxia, Patient-Reported Outcome Measure of Ataxia; SARA, Scale for the assessment and rating of ataxia; UPDRS, Unified Parkinson’s Disease Rating Scale.

### Equipment and supplies

Thirty-eight participants were provided with a study laptop, computer mouse and web camera to perform the study activities, while four participants used a personal computer that met the study criteria. All participants were provided with two GENEActiv wearable sensor devices that collect triaxial accelerometer data at 100 Hz, one for the dominant wrist and one for the dominant ankle (see Supplementary Methods and Supplementary Fig. 1 for additional details).

### Initial study appointment

Over a Zoom video conference, the study coordinator instructed participants on how to activate and properly wear the GENEActiv devices, participants were introduced to the Hevelius computer mouse task, and clinical rating scales were conducted (described below).

### Neurological assessment

All participants completed a neurological assessment via Zoom. An ataxia-specialist neurologist (A.S.G.) performed the Brief Ataxia Rating Scale (BARS)^21^ using the half-point version,^22^ the Scale for the Assessment and Rating of Ataxia (SARA)^23^ and Part III of the Movement Disorder Society-Unified Parkinson Disease Rating Scale (MDS-UPDRS).^24^ The sitting component of SARA and six rigidity components from UPDRS were excluded owing to the rater’s inability to perform/assess these tasks remotely. Thus, the SARA scale score ranged 0–36 instead of 0–40, and MDS-UPDRS Part III ranged 0–108 instead of 0–132. A second ataxia-specialist neurologist (C.D.S.) completed the rating scales from the recorded video, and the average of the two raters was used for analysis. Four participants had 1 missing score, one participant had 2 missing scores, and two participants had 3 missing scores, due to circumstances such as safety concerns, environmental constraints, poor video quality or poor task performance. The primary ataxia rating scale subscores used in analysis were the SARA and BARS gait and SARA and BARS finger–nose–finger, for which there were no missing scores. For SARA, BARS and UPDRS total scores, a linear scaling was applied to individuals with missing scores such that the maximum possible score was aligned across all participants^25^ (Table 1).

### Questionnaires

Participants completed the Patient-Reported Outcome Measure (PROM) questionnaires once at baseline and once at the conclusion of the study. The study feedback survey was completed at the end of the study. The questionnaires included PROM of Ataxia (PROM-Ataxia),^26^ the Dysarthria Impact Scale, Rand 36 Item Short Form Health Survey,^27^ five-level EuroQol 5D (EQ-5D-5L)^28,29^ and neurology quality-of-life (Neuro-QOL) fatigue subscale.^30^ The 70 PROM-Ataxia questions were divided into overlapping subsections for analysis: motor (28), symptoms (25), emotion (10), cognition (7), arm (15) and gait and balance (12) (see Supplementary Table 1).

### Weekly computer mouse task

Participants were asked to complete a computer mouse task (Hevelius) twice per week for 4 weeks (total 8 times). Participants used a mouse to click on targets as soon as they appeared on the screen.^20^ During the first study appointment, participants set the minimum size of the target with a study team member to ensure that the target size was set to a reasonable level of difficulty. During a full session of the computer mouse task, participants performed eight rounds of nine targets per round. The task yields 33 features that describe the participant’s timing, speed and accuracy during the task.^20^ The task also yields composite measures based on previously trained regression and classification models that provide estimates of ataxia and parkinsonism severity and the probability that a participant has ataxia. The outputs from these previously trained models were used in analysis (i.e. models were not trained on the data collected in this study). Descriptions of individual Hevelius features and models can be found in Gajos *et al*.^20^ and Supplementary Table 3.

### Wearable sensor data processing and feature types

Each participant’s wearable sensor data were manually partitioned into day and night segments based on changes in each participant’s daily activity level represented in the accelerometer data.^19,25^ To account for differences in the time of day that sensor recording began across participants, day/night segmentation was started at the onset of the first full night of recording. This produced a maximum of six consecutive 24-h periods of recording. Data analysis focused on daytime segments. Gravity and high-frequency noise were removed from the acceleration time series using a sixth-order Butterworth filter with cut-off frequencies of 0.1 and 20 Hz.^25,31^

**Figure 1:**
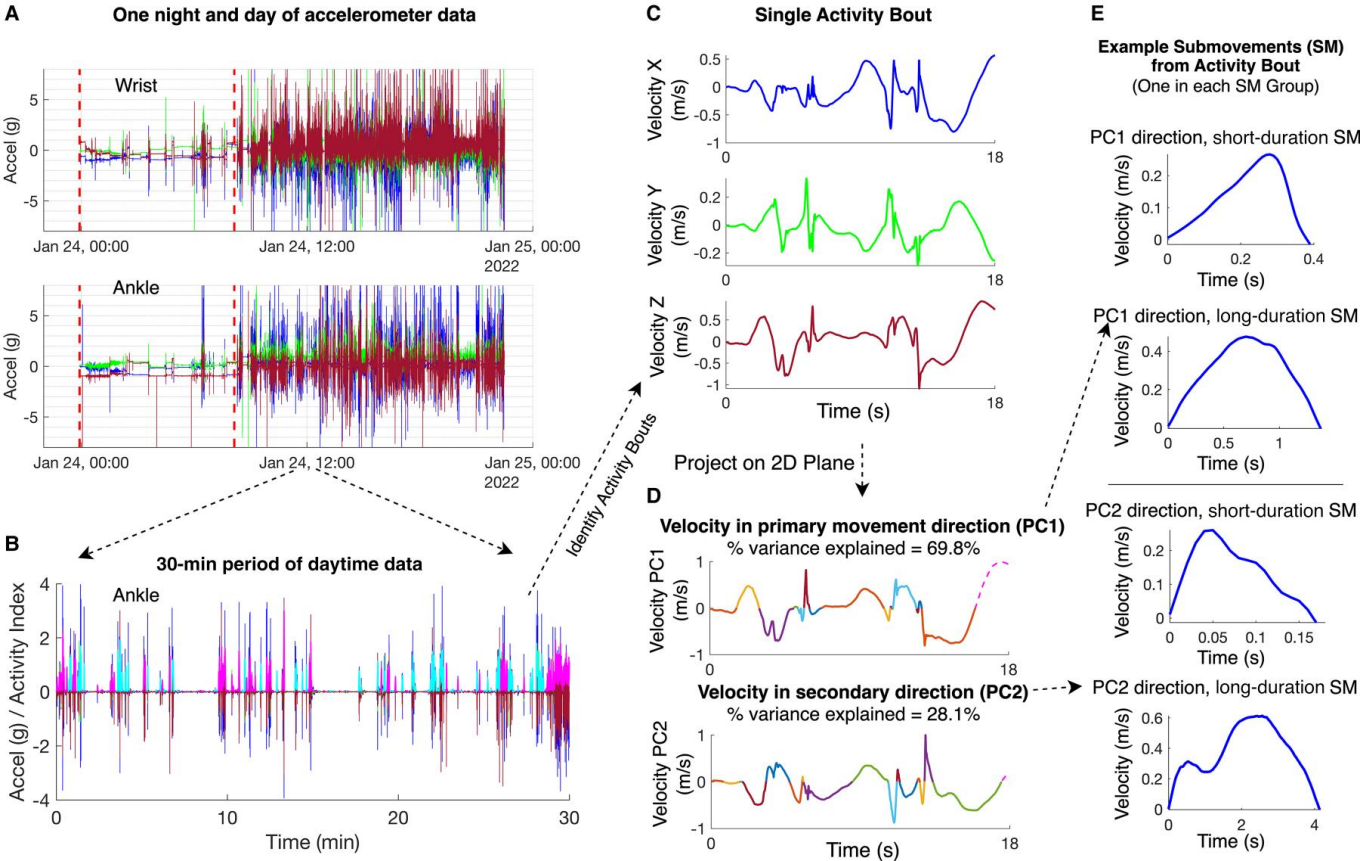
**Overview of wearable sensor data processing steps.** (**A**) One night and day of triaxial accelerometer data shown from a single individual (wrist sensor on top and ankle sensor below). The three lines (blue, green, and red) represent acceleration in the x, y and z directions, respectively. Vertical dotted lines mark the nighttime period. (**B**) A 30‐min segment of triaxial accelerometer data from the ankle sensor. Activity index (normalized to 0–2 range) is plotted for each identified activity bout, alternating between colours (magenta and cyan) to identify each discrete bout. (**C**) A single activity bout in velocity–time space. (**D**) The activity bout projected onto a 2D plane using principal component analysis (PCA), with the primary (PC1) and secondary (PC2) directions of movement plotted. Submovements identified from the activity bout are plotted in different colours[AQ12]. (**E**) Four submovements from the activity bout are shown, one in each of the four submovement groups based on duration (short or long) and direction of movement (PC1 or PC2).

Several classes of features were extracted from daytime ankle and wrist sensor data. These included ‘total power’ in the 0.1- to 5-Hz frequency range and features based on the distribution of ‘activity intensity’ (AI) computed in 1-s time bins, as per previous work from passive wrist sensor data collection in ataxia telangiectasia.^19,25^ Features were also extracted from ‘activity bouts’ and from SMs.^19^Fig. 1 describes how activity bouts and SMs were extracted from continuous accelerometer data collected over a 24-h period. Table 2 provides a description of the 85 features extracted from ankle and wrist sensor data. Based on prior work, single feature analysis was performed on a subset of 26 key features of interest (bolded in Table 2). These included AI mean (one feature), AI entropy (one feature), SM distance (eight features), SM velocity (eight features) and SM acceleration (eight features). Mean and standard deviation were computed over a participant’s SMs for short-duration and long-duration SMs in the primary and secondary directions of planar movement resulting in 2 ∗ 2 ∗ 2 = 8 total features (see Fig. 1 and Table 2).

**Table 2:** Descriptions for each type of wearable sensor feature

Feature level	Feature(s) name	*N*	Description
Activity index	**AI mean**	1	Activity index^32^ was computed for each 1-s window of triaxial accelerometer data over the recording period. ‘Activity intensity (AI) mean’ is the mean activity index value over all daytime activity over the week-long period. ‘Periods of inactivity are excluded from the calculation of AI mean, AI median, AI mode and AI entropy’.^30^
	AI median	1	Median activity intensity over all daytime activity.
	AI mode	1	The most common value (mode) of activity intensity over all daytime activity.
	**AI entropy**	1	The entropy of the distribution of daytime activity intensity.
	% daytime with low AI	1	The percentage of daytime that is spent performing low-intensity movements as previously defined.^30^
	% daytime with moderate AI	1	The percentage of daytime that is spent performing moderate-intensity movements.
	% daytime with high AI	1	The percentage of daytime that is spent performing high-intensity movements.
	% accel in single direction	3	For each 1-s window of movement, principal component analysis was performed on the triaxial accelerometer data to identify the principal direction of acceleration. This feature is the percentage of accelerometer data variance explained by the first principal component direction, averaged over 1-s windows. This measure was computed separately for low AI, moderate AI and high AI 1-s windows resulting in three features.
Spectral	Total power	1	Cumulative power in the 0.1- to 5-Hz frequency band.
Activity bout	Bout acceleration	2	‘Activity bouts’ are continuous periods of activity with durations between 4 and 18 s long based on an activity index threshold.^19^ Bout acceleration is the maximum acceleration in m/s^2^ during an activity bout. ‘*M* and SD are computed over a participant’s activity bouts resulting in two features (applies to bout acceleration and bout jerk)’.
	Bout jerk	2	Bout jerk is the mean jerk (derivative of acceleration) in m/s^3^ during an activity bout.
SM	**SM distance**	8	The distance in meters traveled during a submovement (SM). ‘Mean and standard deviation are computed over a participant’s SMs for short-duration and long-duration SMs in the primary and secondary directions of planar movement resulting in 2 ∗ 2 ∗ 2 = 8 total features (applies to SM distance, velocity, acceleration, jerk and duration)’.
	**SM velocity**	8	The maximum velocity in m/s during a SM.
	**SM acceleration**	8	The maximum acceleration in m/s^2^ during a SM.
	SM jerk	8	The normalized jerk of a SM. This measure is dimensionless and is scaled based on SM duration and SM peak velocity.^20,33,34^
	SM duration	8	The duration of a SM in seconds.
	SM PC1 score	6	The principal component 1 (PC1) score for a submovement. PC1 captures low-frequency characteristics of the SM velocity–time curve (e.g. the SM ‘shape’). The PC1 ‘basis function’ is a single sinusoidal waveform with the peak present in the first half of the submovement.^19^ ‘Mean absolute value, standard deviation and kurtosis are computed for long-duration SMs in the primary and secondary directions of movement resulting in 3 ∗ 2 = 6 total features (applies to SM PC1–5 scores)’.
	SM PC2 score	6	The principal component 2 score for a submovement. Similar to PC1, PC2 captures low-frequency characteristics of the SM velocity–time curve. The PC2 basis function is a single sinusoidal waveform with the peak present in the second half of the submovement.^19^
	SM PC3–5 scores	18	The principal component 3–5 scores for a submovement. PC3–5 scores capture higher frequency characteristics of the SM velocity–time curve. The PC3, PC4 and PC5 basis functions consist of 1.5, 2 and 2.5 sinusoidal cycles, respectively.^19^

Bolded features were preselected for individual feature analysis. AI, activity intensity; *N*, number of features; M, mean; PC, principal component; SD, standard deviation; s, seconds; SM, submovement.

### Ankle sensor regression models

Although single feature analysis was restricted to a subset of 26 features, all 85 ankle sensor features were used as input to regression models trained to estimate clinician-rated ataxia severity and patient-reported function. Given the large number of features relative to the number of participants in the current study, linear regression models with L1 regularization (i.e. lasso regression)^35^ were trained to select a small subset of the input variables. Each feature was *Z*-score transformed prior to model training such that feature value ranges and model weights were comparable. BARS total score was used as the target variable for the ataxia severity estimation model as it offered additional granularity with its half-point scores. PROM-Ataxia was used as the target variable for the motor function estimation model. Leave-one-out cross-validation was used to train and estimate generalization performance of the models/composite measures. Pearson correlation coefficient was used to measure performance, with each model compared with SARA total, SARA gait, BARS total, BARS gait, PROM-Ataxia total and PROM-Ataxia gait subscore.

### Statistical analyses

Statistical analyses were completed in MATLAB (Mathworks, Natick, MA) and SPSS (IBM Corp., Armonk, NY). The Mann–Whitney U-test was used to determine individual feature and age differences between disease and control groups and Cohen’s *d* was used to measure effect size (es). The Mann–Whitney U-test was also used to determine individual feature, age and disease severity differences between female and male participants. The Benjamini–Hochberg method was used to adjust for multiple comparisons, and corrected *P*-values are reported.^36^ Corrected *P*-values < 0.05 were considered significant. Single measure intraclass correlation coefficients (ICCs) were used to determine the test–retest reliability of wrist, ankle and Hevelius features. To evaluate reliability for wrist and ankle features, features were computed from data recorded in Days 1–3 and Days 4–6, separately, and ICCs were computed using a two-way mixed effects model.^37^ The Hevelius task produced features for each of the eight sessions performed over the 4-week period. To evaluate reliability of Hevelius features, the median feature values for the first four sessions and the median values for the last four sessions were computed, and ICCs were computed as described above (if only six sessions were performed, the sessions were split into two three-session groups). Test–retest reliability for questionnaires was similarly evaluated by computing ICCs between Weeks 1 and 4 survey completion. Pearson correlation coefficients and *P*-values were used to evaluate the relationship between ankle sensor, wrist sensor and Hevelius features with ataxia rating scales (SARA and BARS) and patient-reported measures of function (PROM-Ataxia). 95% confidence intervals are reported in parentheses for key comparisons in the main text. Ankle sensor and wrist sensor features were derived from up to a maximum of 6 days of data. For Hevelius, median feature values across up to eight sessions were used in this analysis. As above, the Benjamini–Hochberg method was used to adjust for multiple comparisons for each sensor type.^36^ Pearson correlation coefficients and *P*-values were also used to evaluate relationships between PROMs and ataxia rating scales. As PROMs were performed twice, the mean PROM score was used to compute the correlation coefficient. To avoid inflated correlation values driven by differences between control and ataxia participants, Pearson correlation coefficients were computed using data from ataxia (SCAs and MSA) participants only.

## Results

Demographic and clinical information for participants is shown in Table 1. There were no age differences between ataxia (range: 30–72 years) and control (range: 32–69 years) groups (*P* = 0.86). There were 17 female and 17 male participants in the ataxia group and six female and two male participants in the control group. There were no age (*P* = 0.15) or SARA total score (*P* = 0.42) differences between female and male participants.

### Clinical and patient-reported assessments

Individual-level BARS, SARA, MDS-UPDRS and PROM-Ataxia scores are shown in Table 1, and scores broken down by individual diagnosis are shown in Supplementary Table 2. Four SCA participants were preataxic (two male and two female), defined as having SARA total score < 3 (range: 0.75–1.5),^38,39^ and 11 ataxia participants had a SARA/BARS gait score ≥ 6, indicating the need for a walker.

We found strong pairwise correlations between the remote assessment clinical rating scales (BARS, SARA and MDS-UPDRS). BARS was strongly correlated with both SARA (*r* = 0.97) and MDS-UPDRS (*r* = 0.88). BARS, SARA and MDS-UPDRS demonstrated significant correlations with PROM-Ataxia total score (*r* = 0.75, 0.76 and 0.70, respectively). BARS total demonstrated significant correlations with PROM-Ataxia score subsets of symptoms, motor, arm and gait (*r* = 0.65, 0.80, 0.80 and 0.81, respectively). SARA total also demonstrated significant correlations with PROM-Ataxia symptoms, motor, arm and gait subscores (*r* = 0.67, 0.82, 0.80 and 0.83, respectively). Relationships between ataxia rating scales and PROM-Ataxia are reported in Supplementary Table 4.

Test–retest reliability was high for PROM-Ataxia total score (ICC = 0.95), PROM-Ataxia motor subscore (ICC = 0.95) and PROM-Ataxia symptom subscore (ICC = 0.95) and was moderate for PROM-Ataxia emotion (ICC = 0.79) and cognition (ICC = 0.71) subscores. For the EQ-5D-5L questionnaire, test–retest reliability was high for the mobility (ICC = 0.89), usual activities (ICC = 0.82) and anxiety/depression (ICC = 0.75) subsections. Test–retest was lower for the pain/discomfort (ICC = 0.40) and self-care (ICC = 0.62) sections of the survey. The test–retest properties of patient-reported outcomes are shown in Supplementary Table 5.

### Continuous ankle sensor data

Most ankle SM features were significantly correlated with SARA and BARS total scores and gait subscores, PROM-Ataxia total score and PROM-Ataxia gait subset score (Table 3). There were no ankle sensor-based features that were significantly different between female and male participants.

**Table 3:** Properties of ankle sensor features and models

Sensor	Feature name	Statistic	SM duration group	Direction of motion group	Relationship with SARA (BARS)				Relationship with PROM-Ataxia				Test–retest reliability	Disease versus control	
					Total		Gait subscore		Total		Gait subset				
					*r*	*P*-value	*r*	*P*-value	*r*	*P*-value	*r*	*P*-value	ICC	*P*-value	es
**Ankle (single features)**	SM distance	Mean	Long	PC1	−0.39 (−0.38)	3.0E-02	−0.35 (−0.40)	5.0E-02	−0.60	4.0E-04	−0.54	2.0E-03	0.94	n.s.	-
		Mean	Long	PC2	−0.55 (−0.54)	2.0E-03	−0.51 (−0.55)	3.0E-03	−0.68	4.0E-05	−0.65	9.0E-05	0.95	2.0E-02	1.2
		Mean	Short	PC1	−0.62 (−0.64)	2.0E-04	−0.69 (−0.68)	2.0E-05	−0.47	7.0E-03	−0.53	3.0E-03	0.94	2.0E-02	1.1
		**Mean**	**Short**	**PC2**	**−0.74** (**−0.76)**	**3.0E-06**	**−0.79** (**−0.80)**	**2.0E-07**	**−0**.**62**	**3.0E-04**	**−0**.**66**	**6.0E-05**	**0**.**92**	**5.0E-03**	**1**.**5**
		SD	Long	PC1	-	n.s.	-	n.s.	−0.58	6.0E-04	−0.50	4.0E-03	0.89	n.s.	-
		SD	Long	PC2	−0.43 (−0.44)	2.0E-02	−0.41 (−0.46)	2.0E-02	−0.66	7.0E-05	−0.59	5.0E-04	0.87	n.s.	-
		**SD**	**Short**	**PC1**	**−0.69** (**−0.72)**	**2.0E-05**	**−0.75** (**−0.76)**	**2.0E-06**	**−0**.**60**	**4.0E-04**	**−0**.**64**	**2.0E-04**	**0**.**91**	**2.0E-02**	**1**.**2**
		**SD**	**Short**	**PC2**	**−0.79** (**−0.80)**	**9.0E-07**	**−0.83** (**−0.85)**	**4.0E-08**	**−0**.**74**	**4.0E-06**	**−0**.**75**	**4.0E-06**	**0**.**89**	**5.0E-03**	**1**.**7**
	SM velocity	Mean	Long	PC1	−0.63 (−0.63)	2.0E-04	−0.61 (−0.66)	2.0E-04	−0.77	2.0E-06	−0.72	9.0E-06	0.95	1.0E-02	1.3
		**Mean**	**Long**	**PC2**	**−0.78** (**−0.78)**	**8.0E-07**	**−0.76** (**−0.80)**	**7.0E-07**	**−0**.**80**	**4.0E-07**	**−0**.**81**	**3.0E-07**	**0**.**95**	**1.0E-02**	**1**.**7**
		Mean	Short	PC1	−0.58 (−0.61)	6.0E-04	−0.66 (−0.66)	4.0E-05	−0.39	3.0E-02	−0.45	1.0E-02	0.95	2.0E-02	1.1
		**Mean**	**Short**	**PC2**	**−0.70** (**−0.73)**	**2.0E-05**	**−0.77** (**−0.78)**	**4.0E-07**	**−0**.**55**	**2.0E-03**	**−0**.**61**	**4.0E-04**	**0**.**94**	**5.0E-03**	**1**.**5**
		SD	Long	PC1	−0.41 (−0.42)	2.0E-02	−0.42 (−0.47)	2.0E-02	−0.69	4.0E-05	−0.60	4.0E-04	0.90	n.s.	-
		**SD**	**Long**	**PC2**	**−0.64** (**−0.66)**	**2.0E-04**	**−0.65** (**−0.70)**	**5.0E-05**	**−0**.**80**	**3.0E-07**	**−0**.**75**	**3.0E-06**	**0**.**90**	**8.0E-03**	**1**.**4**
		SD	Short	PC1	−0.60 (−0.64)	3.0E-04	−0.70 (−0.70)	2.0E-05	−0.49	5.0E-03	−0.53	2.0E-03	0.92	2.0E-02	1.2
		**SD**	**Short**	**PC2**	**−0.74** (**−0.77)**	**4.0E-06**	**−0.82** (**−0.84)**	**4.0E-08**	**−0**.**69**	**4.0E-05**	**−0**.**71**	**2.0E-05**	**0**.**89**	**5.0E-03**	**1**.**7**
	SM acceleration	Mean	Long	PC1	−0.70 (−0.74)	2.0E-05	−0.74 (−0.76)	2.0E-06	−0.57	8.0E-04	−0.57	8.0E-04	0.96	5.0E-03	1.6
		**Mean**	**Long**	**PC2**	**−0.78** (**−0.80)**	**7.0E-07**	**−0.81** (**−0.83)**	**6.0E-08**	**−0**.**61**	**4.0E-04**	**−0**.**65**	**9.0E-05**	**0**.**94**	**5.0E-03**	**1**.**8**
		Mean	Short	PC1	−0.46 (−0.50)	8.0E-03	−0.56 (−0.55)	7.0E-04	-	n.s.	-	n.s.	0.96	2.0E-02	1.0
		Mean	Short	PC2	−0.58 (−0.63)	6.0E-04	−0.68 (−0.68)	2.0E-05	−0.40	3.0E-02	−0.46	8.0E-03	0.95	9.0E-03	1.3
		SD	Long	PC1	−0.47 (−0.52)	7.0E-03	−0.56 (−0.58)	7.0E-04	−0.38	3.0E-02	−0.36	4.0E-02	0.97	2.0E-02	1.3
		SD	Long	PC2	−0.62 (−0.65)	2.0E-04	−0.71 (−0.72)	7.0E-06	−0.49	5.0E-03	−0.50	4.0E-03	0.92	4.0E-03	1.5
		SD	Short	PC1	-	n.s.	−0.42 (−0.41)	2.0E-02	-	n.s.	-	n.s.	0.95	n.s.	-
		SD	Short	PC2	−0.44 (−0.49)	2.0E-02	−0.57 (−0.58)	7.0E-04	−0.38	4.0E-02	−0.40	3.0E-02	0.90	2.0E-02	1.2
	AI	**Mean**	**N/A**	**N/A**	**−0.67** (**−0.66)**	**4.0E-05**	**−0.73** (**−0.76)**	**3.0E-06**	**−0**.**65**	**1.0E-04**	**−0**.**68**	**4.0E-05**	**0**.**88**	**6.0E-03**	**1**.**5**
		**Entropy**	**N/A**	**N/A**	**−0.72** (**−0.72)**	**8.0E-06**	**−0.78** (**−0.80)**	**3.0E-07**	**−0**.**65**	**9.0E-05**	**−0**.**70**	**2.0E-05**	**0**.**93**	**8.0E-03**	**1**.**4**
**Ankle (models)**	**Ataxia severity prediction model**				**0.82 (0.83)**	**4.0E-09**	**0.84 (0.88)**	**4.0E-10**	**0.81**	**9.0E-09**	**0.81**	**7.0E-09**	**0.95**	4.0E-04^*^	**1.8**
	**Self-reported function prediction model**				**0.82 (0.82)**	**3.0E-09**	**0.82 (0.86)**	**4.0E-09**	**0.83**	**2.0E-09**	**0.83**	**2.0E-09**	**0.94**	6.0E-04^*^	**1.6**

Relationships with ataxia rating scales and patient-reported function, test–retest reliability and disease versus control statistics are provided. Key features/models are bolded. Features that were significantly different between preataxic individuals and controls are marked in the disease versus control p-value column with an (*).. Relationships that are not significant are labelled as ‘n.s.’ AI, activity intensity; BARS, Brief Ataxia Rating Scale; ICC, intraclass correlation coefficient; es, effect size; PC, Principal Component; PROM-Ataxia, Patient-Reported Outcome Measure of Ataxia; *r*, Pearson correlation coefficient; SARA, Scale for the assessment and rating of ataxia; SD, standard deviation; SM, submovement.

### Ankle SM distance

For ankle SM distance features, short-duration SMs in the direction orthogonal to the primary direction of movement [i.e. principal component 2 (PC2) direction] were most strongly related to SARA, BARS and PROM-Ataxia (see bolded rows in Table 3). Mean distance of this SM group was strongly negatively correlated with SARA total [*r* = −0.74 (−0.54 to −0.86)] and SARA gait subscore [*r* = −0.79 (−0.61 to −0.89)] and was moderately correlated with PROM-Ataxia total [*r* = −0.62 (−0.35 to −0.79)] and PROM-Ataxia gait subscore [*r* = −0.66 (−0.42 to −0.82)]. Variance of distances of short-duration SMs in the PC2 direction of movement were strongly negatively correlated with SARA total, SARA gait, PROM-ataxia total and PROM-Ataxia gait [*r* = −0.79 (−0.61 to −0.89), −0.83 (−0.68 to −0.91), −0.74 (−0.54 to −0.86) and −0.75 (−0.56 to −0.87), respectively]. These two SM distance features had high test–retest reliability across the first and second half of the week of data collection (ICC = 0.89–0.92) and were significantly different between ataxia and control participants [es = 1.5–1.7, *P* < 0.005]. Thus, SM distances were smaller and less variable in individuals with ataxia and became progressively smaller with reduced self-reported function and increased ataxia severity, especially for short-duration SMs orthogonal to the primary direction of movement.

### Ankle SM peak velocity

**Figure 2:**
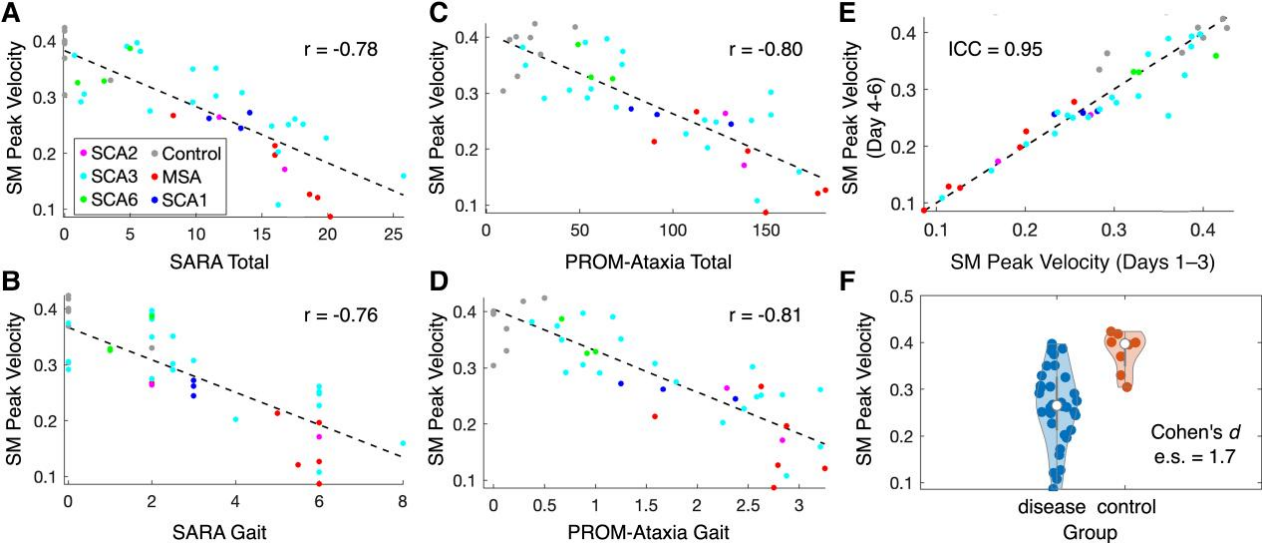
**Properties of a single ankle submovement feature: peak velocity of long-duration submovements in the secondary direction of movement (*n* = 42).** (**A** and **B**) Relationship of the feature with SARA total score and gait subscore. (**C** and **D**) Relationship of the feature with PROM-Ataxia total score and gait subscore. (**E**) Test–retest reliability of the feature. (**F**) Disease versus control violin plot. PROM-Ataxia, Patient-Reported Outcome Measure of Ataxia; SARA, Scale for the Assessment and Rating of Ataxia.

SM peak velocity features also demonstrated that SMs in the PC2 direction (orthogonal to the primary direction of movement) were most strongly related to SARA and PROM-Ataxia. SM peak velocity was informative for both long and short-duration SM groups (Table 3). Mean peak velocity of the long-duration SM group in the PC2 direction was highly negatively correlated with SARA total [*r* = −0.78 (−0.60 to −0.88)], SARA gait subscore [*r* = −0.76 (−0.57 to −0.87)], PROM-Ataxia total [*r* = −0.80 (−0.63 to −0.90)] and PROM-Ataxia gait subscore [*r* = −0.81 (−0.64 to −0.90)]. This feature also showed very high test–retest reliability (ICC = 0.95) and strongly distinguished ataxia and control groups (es = 1.7, *P* < 0.01). Scatter plots for these relationships are shown in Fig. 2. Variance in peak velocities of both long and short-duration SMs in the PC2 direction of movement showed similar properties (Table 3). Thus, SM peak velocities became progressively smaller and less variable with decreasing self-reported function and increased ataxia severity, especially for SMs orthogonal to the primary direction of movement.

### Ankle SM peak acceleration

SM peak acceleration features were informative for longer duration SMs in the PC2 direction, but less so for shorter duration SMs. Mean peak acceleration of this SM group was strongly negatively correlated with SARA total [*r* = −0.78 (−0.59 to −0.88)] and SARA gait subscore [*r* = −0.81 (−0.65 to −0.90)] and moderately correlated with PROM-Ataxia total [*r* = −0.61 (−0.34 to −0.78)] and PROM-Ataxia gait subscore [*r* = −0.65 (−0.40 to −0.81)]. This feature showed high test–retest reliability (ICC = 0.94) and strongly distinguished ataxia and control groups (es = 1.8, *P* < 0.005). All four SM peak acceleration variability features were significantly lower in preataxic individuals (*n* = 4) compared to controls (*n* = 7) with SARA total score < 3, although they did not remain significant after correction for multiple comparisons. Out of all 26 individual ankle sensor features, these were the only four that were significantly different between preataxic individuals and controls prior to correction for multiple comparisons.

### Ankle AI

AI mean and entropy were negatively correlated with SARA total [*r* = −0.67 (−0.43 to −0.82) and −0.72 (−0.50 to −0.85), respectively], SARA gait subscore [*r* = −0.73 (−0.52 to −0.86) and −0.78 (−0.61 to −0.89)], PROM total [*r* = −0.65 (−0.39 to −0.81) and −0.65 (−0.40 to −0.81)] and PROM gait subscore [*r* = −0.68 (−0.45 to −0.83) and −0.70 (−0.47 to −0.84)]. The two AI-based features showed high test–retest reliability (ICC = 0.88, 0.93) and were different between ataxia and control participants (es = 1.5, 1.4, *P* < 0.01). These findings indicate that ankle movements were progressively less intense with a narrower range of intensity levels as disease severity increased among participants in the study.

### Ankle regression models

Two separate regression models were trained, one to estimate ataxia severity and one to estimate self-reported function, based on the full set of 85 ankle sensor features. As shown in Table 3, the ataxia severity prediction model correlated strongly with SARA total [*r* = 0.82 (0.66–0.91)], SARA gait [*r* = 0.84 (0.71–0.92)], BARS total [*r* = 0.83 (0.68–0.91)], BARS gait [*r* = 0.88 (0.77–0.94)], PROM-Ataxia total [*r* = 0.81 (0.64–0.90)] and PROM-Ataxia gait [*r* = 0.81 (0.65–0.90)]. The model had very high test–retest reliability (ICC = 0.95) and strongly distinguished ataxia and control participants (es = 1.8, *P* < 0.001). Both models also were significantly different between preataxic and control participants with SARA total score < 3 (es = 1.4–1.6, *P* < 0.05). Across all cross-validation folds, the model drew information primarily from only four features: variance in the distance of short-duration SMs in the PC2 direction, mean peak velocity of long-duration SMs in the PC2 direction, mean jerk during activity bouts and percent of acceleration data variance explained in a single direction for high AI 1-s windows (see Table 2). The first two selected features were expected based on the single feature analysis described above. The latter two features, which were not *a priori* included in individual feature analysis, indicated that individuals with ataxia had progressively lower mean jerk during activity bouts and a progressively higher percent of triaxial (i.e. 3D) acceleration variance explained by a single direction, as disease severity increased. These two features suggest that natural ankle movements become less powerful and less flexible as disease progresses. The four informative features were selected in 100% of cross-validation folds with average model coefficients of −1.49, −1.06, −1.33 and 0.81, respectively. Only three other features were selected in any cross-validation folds; two were selected in 2% of folds, and one was selected in 12% of folds. The second regression model that was explicitly trained to estimate self-reported function generated outputs with similar properties (Table 3); however, more features were selected across all cross-validation folds (27) with an average of 9.5 features selected per fold.

### Continuous wrist sensor data

The majority of wrist SM distance, velocity and acceleration features were significantly correlated with SARA, BARS and PROM-Ataxia (Supplementary Table 6). The observed relationships with patient-reported function and ataxia severity were less strong compared to ankle SMs: across all wrist SM features, the strongest correlations with each clinical and patient-reported score were −0.64 (−0.39 to −0.81) with SARA total, −0.46 (−0.14 to −0.69) with SARA arm, −0.56 (−0.27 to −0.75) with BARS arm, −0.66 (−0.42 to −0.82) with PROM-Ataxia total and −0.68 (−0.44 to −0.83) with PROM-Ataxia arm. Correlations between wrist sensor features and BARS finger–nose–finger score were stronger and more often statistically significant than correlations with SARA finger–nose–finger score. As with ankle SMs, wrist SM distance, peak velocity and peak acceleration became progressively smaller and less variable with reduced self-reported function and increased ataxia severity. There were no wrist sensor-based features that were significantly different between female and male participants. Although correlations with clinical scales were lower for the wrist sensor compared with the ankle sensor, many wrist movement features demonstrated very high test–retest reliability (Supplementary Table 6). This indicates that reliable information is obtained from the wrist sensor, but it differs substantially from information captured in clinical scales. Longitudinal data are needed to determine if wrist sensor information sensitively captures disease change over time as seen in ataxia telangiectasia.^19^

### Hevelius computer mouse task data

There were no Hevelius computer mouse task features that were significantly different between female and male participants. Most Hevelius features were significantly correlated with SARA and BARS total scores and arm subscores, PROM-Ataxia total score and PROM-Ataxia arm subset score (Table 4). Individuals with ataxia took longer and had more variability in the time to perform each trial of the task. The coefficient of variation (CV) of movement time was strongly positively correlated with SARA total [*r* = 0.85 (0.72–0.92), respectively], SARA arm [*r* = 0.66 (0.41–0.82)], BARS arm [*r* = 0.73 (0.52–0.86)], PROM-Ataxia total [*r* = 0.71 (0.48–0.84)] and PROM-Ataxia arm [*r* = 0.73 (0.52–0.86)]. The mean and CV of movement time also showed very high test–retest reliability (ICC = 0.99 and 0.94, respectively) and strongly distinguished between ataxia and control participants (es = 2.0 and 1.7, *P* < 0.002). The number of pauses and duration of the longest pause were increased in individuals with ataxia and showed similarly strong correlations with ataxia rating scales and self-reported function along with high test–retest reliability. Individuals with ataxia had higher normalized jerk during their mouse movements and demonstrated reduced accuracy of movements as reflected by larger distances to the target remaining after the main SM and more target re-entries. The number of movement direction changes was the only feature that was significantly different between preataxic (*n* = 4) and control (*n* = 7) participants with SARA total score < 3; however, this did not remain significant after correction for multiple comparisons.

**Table 4:** Properties of Hevelius computer mouse task features and models

Sensor	Feature name	Relationship with SARA (BARS)				Relationship with PROM-Ataxia				Test–retest reliability	Disease versus control	
		Total		Arm subscore (FNF)		Total		Arm subset				
		*r*	*P*-value	*r*	*P*-value*	*r*	*P*-value	*r*	*P*-value	ICC	*P*-value	es
**Hevelius (single features)**	**Movement time**	**0.84** (**0.83)**	**9.0E-09**	**0.61** (**0.73)**	**(6.0E-06)**	**0**.**67**	**2.0E-04**	**0**.**68**	**4.0E-05**	**0**.**99**	**2.0E-03**	**2**.**0**
	**Movement time (CV)**	**0.85** (**0.86)**	**8.0E-09**	**0.66** (**0.73)**	**(6.0E-06)**	**0**.**71**	**2.0E-04**	**0**.**73**	**3.0E-05**	**0**.**94**	**2.0E-03**	**1**.**7**
	**Execution time**	**0.83** (**0.81)**	**3.0E-08**	**0.56** (**0.69)**	**(3.0E-05)**	**0**.**67**	**2.0E-04**	**0**.**68**	**4.0E-05**	**0**.**99**	**2.0E-03**	**1**.**9**
	**Execution time (CV)**	**0.82** (**0.83)**	**2.0E-08**	**0.69** (**0.75)**	**(4.0E-06)**	**0**.**68**	**2.0E-04**	**0**.**72**	**3.0E-05**	**0**.**92**	**9.0E-03**	**1**.**3**
	Execution time without pauses	0.76 (0.74)	8.0E-07	0.46 (0.59)	(6.0E-04)	0.63	3.0E-04	0.60	5.0E-04	0.97	2.0E-03	1.8
	**Execution time without pauses (CV)**	**0.79** (**0.82)**	**9.0E-08**	**0.73** (**0.77)**	**(2.0E-06)**	**0**.**65**	**2.0E-04**	**0**.**72**	**3.0E-05**	**0**.**84**	**2.0E-03**	**1**.**9**
	Verification time	0.45 (0.47)	9.0E-03	0.47 (0.46)	(1.0E-02)	0.43	2.0E-02	0.40	3.0E-02	0.89	n.s.	-
	Verification time (SD)	0.75 (0.76)	2.0E-06	0.66 (0.73)	(6.0E-06)	0.59	6.0E-04	0.58	8.0E-04	0.96	2.0E-03	1.7
	**Number of pauses**	**0.82** (**0.82)**	**3.0E-08**	**0.69** (**0.78)**	**(2.0E-06)**	**0**.**62**	**4.0E-04**	**0**.**69**	**4.0E-05**	**0**.**97**	**2.0E-03**	**2**.**0**
	**Duration of longest pause**	**0.82** (**0.81)**	**3.0E-08**	**0.69** (**0.78)**	**(1.0E-06)**	**0**.**61**	**4.0E-04**	**0**.**68**	**5.0E-05**	**0**.**97**	**2.0E-03**	**2**.**0**
	Max speed	-	n.s.	-	n.s.	-	n.s.	-	n.s.	0.95	n.s.	-
	Max speed (CV)	0.72 (0.76)	4.0E-06	0.54 (0.62)	(3.0E-04)	0.52	3.0E-03	0.59	7.0E-04	0.85	2.0E-03	1.8
	Max acceleration	-	n.s.	-	n.s.	-	n.s.	-	n.s.	0.95	n.s.	-
	Max acceleration (CV)	0.56 (0.60)	9.0E-04	0.44 (0.49)	(6.0E-03)	0.37	4.0E-02	0.43	2.0E-02	0.80	5.0E-03	1.3
	**Normalized jerk**	**0.80** (**0.80)**	**7.0E-08**	**0.56** (**0.67)**	**(5.0E-05)**	**0**.**67**	**2.0E-04**	**0**.**69**	**3.0E-05**	**0**.**98**	**3.0E-03**	**1**.**9**
	Normalized jerk without pauses	0.75 (0.75)	2.0E-06	0.46 (0.59)	(6.0E-04)	0.61	4.0E-04	0.62	3.0E-04	0.97	2.0E-03	1.9
	Click duration	0.75 (0.68)	2.0E-06	0.51 (0.64)	(2.0E-04)	0.53	3.0E-03	0.47	7.0E-03	0.96	2.0E-03	1.6
	Click duration (SD)	0.67 (0.61)	3.0E-05	0.40 (0.52)	(4.0E-03)	0.49	6.0E-03	0.44	2.0E-02	0.82	2.0E-03	1.3
	Movement direction changes	0.45 (0.47)	9.0E-03	-	n.s.	0.37	4.0E-02	0.44	2.0E-02	0.93	3.0E-03	1.8
	Orthogonal direction changes	0.61 (0.60)	3.0E-04	0.34 (0.48)	(8.0E-03)	0.50	5.0E-03	0.56	2.0E-03	0.95	5.0E-03	1.4
	Task axis crossings	0.47 (0.49)	7.0E-03	0.28 (0.38)	(3.0E-02)	0.42	2.0E-02	0.48	6.0E-03	0.89	2.0E-03	1.7
	Max deviation from task axis	0.55 (0.55)	2.0E-03	0.28 (0.40)	(3.0E-02)	0.48	6.0E-03	0.50	4.0E-03	0.93	n.s.	-
	Movement error	0.59 (0.59)	5.0E-04	0.33 (0.43)	(2.0E-02)	0.54	3.0E-03	0.56	2.0E-03	0.94	n.s.	-
	Movement offset	0.58 (0.58)	5.0E-04	0.31 (0.41)	(3.0E-02)	0.49	5.0E-03	0.48	6.0E-03	0.85	n.s.	-
	Movement variability	0.56 (0.56)	1.0E-03	0.30 (0.41)	(3.0E-02)	0.50	5.0E-03	0.52	3.0E-03	0.94	n.s.	-
	**Distance from target at end of main SM**	**0.79** (**0.81)**	**9.0E-08**	**0.59** (**0.69)**	**(3.0E-05)**	**0**.**66**	**2.0E-04**	**0**.**69**	**4.0E-05**	**0**.**95**	**2.0E-03**	**2**.**2**
	**Target re-entries**	**0.71** (**0.73)**	**6.0E-06**	**0.55** (**0.64)**	**(2.0E-04)**	**0**.**64**	**3.0E-04**	**0**.**71**	**3.0E-05**	**0**.**93**	**2.0E-03**	**1**.**9**
	Click slip	0.45 (0.42)	9.0E-03	-	n.s.	-	n.s.	-	n.s.	0.86	2.0E-02	1.2
	Fraction distance covered in main SM	0.42 (0.48)	2.0E-02	-	n.s.	0.41	2.0E-02	0.49	6.0E-03	0.82	3.0E-02	1.0
	Fraction of main SM spent accelerating	0.50 (0.48)	3.0E-03	0.39 (0.39)	(3.0E-02)	0.51	4.0E-03	0.48	6.0E-03	0.84	n.s.	-
	Number of submovements	0.55 (0.58)	2.0E-03	0.53 (0.55)	(2.0E-03)	0.46	8.0E-03	0.56	2.0E-03	0.96	2.0E-02	1.1
	Main submovement	0.56 (0.60)	9.0E-04	0.32 (0.39)	(3.0E-02)	0.51	4.0E-03	0.60	5.0E-04	0.83	n.s.	-
	Noise to force ratio	0.74 (0.73)	2.0E-06	0.53 (0.63)	(2.0E-04)	0.60	5.0E-04	0.54	2.0E-03	0.94	2.0E-03	1.7
**Hevelius (models)**	**BARS total prediction model**	**0.85** (**0.84)**	**2.0E-10**	**0.58** (**0.71)**	**(3.0E-06)**	**0**.**67**	**2.0E-05**	**0**.**70**	**5.0E-06**	**0**.**98**	**9.0E-05**	**2**.**2**
	**BARS arm prediction model**	**0.87** (**0.85)**	**4.0E-11**	**0.59** (**0.72)**	**(2.0E-06)**	**0**.**69**	**8.0E-06**	**0**.**70**	**4.0E-06**	**0**.**98**	**2.0E-04**	**2**.**1**
	**UPDRS total prediction model**	**0.88** (**0.86)**	**5.0E-12**	**0.65** (**0.75)**	**(4.0E-07)**	**0**.**73**	**1.0E-06**	**0**.**72**	**2.0E-06**	**0**.**99**	**4.0E-04**	**1**.**8**
	**UPDRS arm prediction model**	**0.80** (**0.79)**	**2.0E-08**	**0.62** (**0.70)**	**(4.0E-06)**	**0**.**64**	**5.0E-05**	**0**.**63**	**6.0E-05**	**0**.**98**	**3.0E-04**	**1**.**8**
	**Pairwise comp total prediction model**	**0.83** (**0.82)**	**2.0E-09**	**0.60** (**0.71)**	**(3.0E-06)**	**0**.**68**	**2.0E-05**	**0**.**71**	**3.0E-06**	**0**.**97**	3.0E-05*	**2**.**5**
	**Pairwise comp arm prediction model**	**0.81** (**0.81)**	**5.0E-09**	**0.66** (**0.75)**	**(3.0E-07)**	**0**.**63**	**7.0E-05**	**0**.**66**	**2.0E-05**	**0**.**96**	2.0E-05*	**2**.**7**
	Ataxia versus controls prediction model	0.73 (0.71)	2.0E-06	0.53 (0.65)	(4.0E-05)	0.48	5.0E-03	0.53	2.0E-03	0.95	2.0E-05*	4.0

Relationships with ataxia rating scales and patient-reported function, test–retest reliability, and disease versus control statistics are provided. Key features/models are bolded. Features that were significantly different between preataxic individuals and controls are marked in the disease versus control *P*-value column with an (*). Relationships that are not significant are labelled as ‘n.s.’. The *P*-values reported for the relationships with the ataxia rating scale arm subscore are for the BARS arm subscore as these relationships were stronger than the SARA arm subscore relationships. BARS, Brief Ataxia Rating Scale; CV, coefficient of variation; es, effect size; FNF, finger–nose–finger; ICC, intraclass correlation coefficient; PROM-Ataxia, Patient-Reported Outcome Measure of Ataxia; *r*, Pearson correlation coefficient; SARA, scale for the assessment and rating of ataxia; SD, standard deviation; SM, submovement; UPDRS, Unified Parkinson’s Disease Rating Scale.

**Figure 3:**
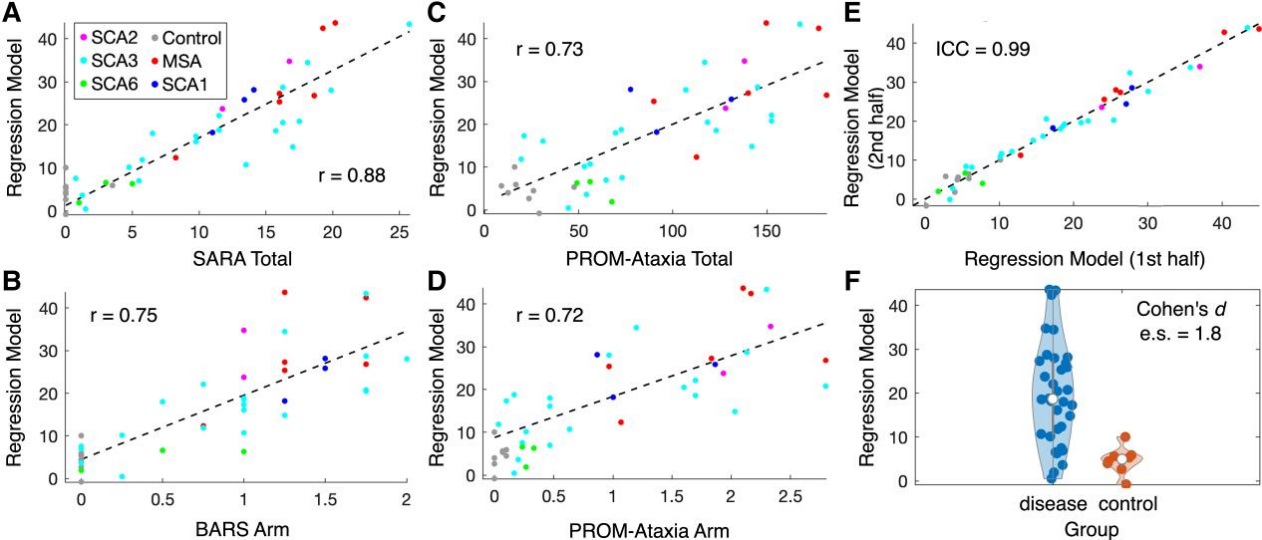
**Properties of a Hevelius composite model: regression model previously trained to estimate parkinsonism severity (*n* = 42).** This model, which was trained to estimate parkinsonism severity as represented by UPDRS Part III, demonstrated particularly strong relationships with ataxia rating scales, patient-reported function, and had high test–retest reliability. (**A** and **B**) Relationship of the model with SARA total score and BARS arm subscore. Hevelius task features were consistently more strongly associated with BARS finger–nose–finger score than SARA finger–nose–finger; thus, the relationship with BARS arm is shown (see Table 4). (**C** and **D**) Relationship of the model with PROM-Ataxia total score and arm subscore. (**E**) Test–retest reliability of the model. (**F**) Disease versus control violin plot. BARS, Brief Ataxia Rating Scale; PROM-Ataxia, Patient-Reported Outcome Measure of Ataxia; SARA, Scale for the Assessment and Rating of Ataxia; UPDRS, unified Parkinson’s Disease Rating Scale.

The previously trained regression model for estimating UPDRS Part III^20^ showed particularly strong correlations with SARA total [*r* = 0.88 (0.78–0.94)], BARS arm [*r* = 0.75 (0.55–0.87)], PROM-Ataxia total [*r* = 0.73 (0.52–0.86)] and PROM-Ataxia arm [*r* = 0.72 (0.50–0.85)]. This model had an ICC of 0.99 and differentiated ataxia and control participants with an es of 1.8 (Fig. 3). The previously trained pairwise comparisons severity estimation models and the classification model^20^ were significantly different between preataxic individuals and control participants with SARA total score < 3 (es = 1.6–2.0, *P* < 0.03). The pairwise comparisons severity estimation models also strongly differentiated all ataxia and control participants (es = 2.5–2.7) and had strong relationships with ataxia severity and self-reported function (Supplementary Fig. 2). Regression model parameters are shown in Supplementary Table 3.

### Study feedback survey

Participants completed the feedback survey on the last day of the study. Seventy-six percent (32/42) of participants reported that they would be willing to complete the full assessment again, 14% (6/42) of participants reported that they would be willing to complete a shorter version of the assessment, 7% (3/42) were unsure and 2% (1/42) reported that they probably would not be willing to complete the assessment again. 51.9% (14/29) of ataxia participants who reported unsteadiness responded that they thought the wearable sensor was able to capture their unsteadiness. Three individuals noted that they were unsure what the wearable sensors were measuring. Thirty-five percent (8/23) of ataxia participants who reported a lack of coordination and/or fatigue responded that they thought the computer mouse task was able to capture these symptoms.

## Discussion

We have shown that digital devices used entirely at home can characterize and quantify self-reported motor function and ataxia with high accuracy and high reliability. In particular, a regression model based on continuous at-home ankle accelerometer data produced a motor measure that strongly correlated with ataxia rating scale total and gait scores (*r* = 0.82–0.88), strongly correlated with self-reported overall and gait function (*r* = 0.81), had high test–retest reliability (ICC = 0.95) and distinguished ataxia and control participants, including preataxic individuals. A regression model based on at-home computer mouse task performance produced a motor measure that also strongly correlated with ataxia rating scale total (*r* = 0.86–0.88) and arm scores (*r* = 0.65–0.75), correlated well with self-reported overall and arm function scores (*r* = 0.72–0.73) and had high test–retest reliability (ICC = 0.99). These data demonstrate that the two assessment technologies provide meaningful and reliable measures of motor function in degenerative ataxias and have population-level sensitivity to disease change. Both tools should be evaluated longitudinally in natural history studies to assess individual-level sensitivity to disease progression over time.

### Ankle SM characteristics in ataxia

The ankle sensor used in this study was worn continuously for 1 week and did not require that participants perform a specific motor task. Interpretation of passively collected accelerometer data can be challenging without knowledge of the specific behaviours being performed. To address this challenge, data analysis focused on characterizing motor primitives called SMs, extracted automatically from accelerometer data during natural behaviour.^19^ There is evidence that motor control is achieved by combining elementary SMs to compose voluntary motor behaviours.^40-43^ The concept of movement composition from SMs is of particular relevance in cerebellar ataxias where movements are observed to become segmented or decomposed into constituent parts,^44^ potentially due to dyssynchrony of the movement components^45,46^ or as a compensatory strategy to maximize terminal movement accuracy.^47-49^ Thus, SM-level analysis provides a mechanism to quantify motor impairment—specifically decomposition of movement—without needing to identify specific types of motor behaviours. We found that ankle SM distance, peak velocity and peak acceleration were smaller in ataxia participants compared to controls and became progressively smaller and less variable as self-reported function decreased and ataxia severity increased. SMs in the plane orthogonal to the primary direction of motion were highly reflective of motor function and ataxia severity; more so than SMs in the primary direction of motion. All four SM acceleration variance measures showed decreased variability in peak acceleration in preataxic individuals compared to controls, although this did not remain significant after correction for multiple comparisons. This pattern of smaller, less powerful, and less flexible SMs in ataxia is consistent with recent descriptions of ankle SMs in adults with ataxia during a gait task,^50^ arm SMs in individuals with ataxia during reaching tasks^51-53^ and wrist SMs in a paediatric genetic ataxia (ataxia telangiectasia) during natural behaviour.^19^ These SM changes reflect the hallmark characteristic of the ataxia phenotype that movements become segmented or decomposed into smaller movements.^44^ The wrist sensor data presented here also demonstrated progressively smaller SM distance, peak velocity and peak acceleration, with high test–retest reliability. The SM changes observed were similar to changes seen in healthy older individuals^54^ and, as expected, were in the opposite direction of the changes seen during infant motor development^55^ and stroke recovery.^56^ Thus, characterization of SMs during natural behaviour may also be a useful basis for motor assessments in other conditions affecting movement.

### Computer mouse task characteristics in ataxia

The Hevelius computer mouse task was performed twice per week for 4 weeks (8 times total), requiring the participant to use a mouse to click targets on the screen for 1.3–9.0 (mean = 3.8) minutes each time. Individuals with ataxia took longer to perform each trial and had longer and more pauses, and their mouse movements were less smooth and less accurate. The number of movement direction changes were increased in preataxic individuals compared to controls, although this did not remain significant after correction for multiple comparisons. These characteristics are consistent with clinical characterization of the ataxia motor phenotype,^44,57^ prior ‘in-clinic’ evaluation of computer mouse movements in individuals with ataxia^20^ and evaluation of arm movements in ataxia using other digital technologies.^32,58^ All previously trained Hevelius regression models showed strong relationships with ataxia rating scales and patient-reported function. The models trained based on pairwise comparisons between individuals with ataxia and parkinsonism^20^ were also able to significantly differentiate preataxic and control participants. Interestingly, the regression model previously trained to estimate UPDRS Part III showed the best performance in estimating ataxia severity and participant function. This model strongly weighted mouse movement and click features including task axis crossings, execution time, fraction of the main SM spent accelerating, number of SMs, max speed, click duration variability and click slip (model weights are shown in Supplementary Table 3). The features included in this model have relevance for both parkinsonism and ataxia phenotypes and highlight the utility of creating composite motor measures, which have the potential to be more accurate and reliable than single features.

### Reliability of wearable sensor and Hevelius measures

We found that the vast majority of ankle and wrist sensor features had very high test–retest reliability when comparing data from Days 1–3 with Days 4–6. The two composite regression models trained on ankle data had ICCs of 0.95 and 0.94. The high reliability of SM features and models is driven in part by the aggregation of information over thousands of motor primitives collected from many different behaviours over multiple days. This enables the measures to account for diurnal and daily fluctuations in the disease state. Reliability is expected to be even higher when using data from an entire week.

The Hevelius computer mouse task also showed very high test–retest reliability when comparing the median performance on the task during the first 2 weeks of the study with the last 2 weeks. Each session of Hevelius integrates information over 64 trials and median performance over a few sessions (three to four) produced highly reliable motor measurements with an ICC of 0.99 for the UPDRS regression model.

### Ecological validity of ankle sensor measures

Continuous recording of movement using wearable sensors directly captures daily motor behaviours and has the potential to produce measures that closely reflect motor functions that are meaningful to individuals with ataxia. Recent studies in adult ataxias have used a sophisticated three-sensor system (two ankle sensors and one lumbar sensor) to assess gait^33^ and turn^34^ characteristics during a several-hour, unsupervised period at home, with ataxia participants instructed to include at least a 30-min walk (unassisted by walking aids) alongside their usual everyday activities. In these studies, specific gait characteristics including lateral step deviation and spatial step variability were strongly correlated with clinical ataxia severity as measured on SARA gait and posture subscore, with a Spearman *ρ* of 0.76.^33^ Furthermore, turn characteristics including lateral velocity change and outward acceleration strongly correlated with clinical ataxia severity (*ρ* = 0.79 with SARA total score) and also correlated well with patient-reported balance confidence on the activity-specific balance confidence scale^59^ (*ρ* = 0.66).^34^

Our work builds upon these real-life quantitative phenotyping studies and demonstrates that a single consumer-grade ankle sensor worn continuously for multiple days, without guidelines or restrictions on behaviour, can produce measures that closely reflect patient-reported function. The ankle sensor regression models, based on a small number of interpretable SM characteristics, strongly correlated with patient-reported function, as measured on PROM-Ataxia total and gait subset, with correlation coefficients of 0.81 and 0.83. These correlations with PROM-Ataxia were higher than clinical ataxia rating scale correlations with PROM-Ataxia (SARA: 0.76; BARS: 0.75) and higher than the Hevelius regression model’s correlation with PROM-Ataxia (0.73). Correlation of the ankle sensor-based model with SARA total score was also high with a correlation coefficient of 0.82. These observations are consistent with the intuition that information derived from the individual’s own selection of behaviours—their typical and natural daily behaviour—can accurately and, perhaps most strongly, reflect the individual’s own perception of their daily function.

### Feasibility and clinical applicability

Participants in the study included individuals who were preataxic as well as individuals who used assistive devices such as walkers. Thus the assessment tools were informative and feasible across a wide range of disease stages. While the existing regression models demonstrate strong performance across the spectrum of disease severity, additional models could be trained in the future that are tailored for a specific goal (e.g. estimation of severity in very early disease states).

The motor assessment tools utilized relatively inexpensive and easy-to-use devices. The wearable sensor is commercially available and costs less than $350. The only requirements for the Hevelius computer mouse task are that the laptop or desktop computer has at least a 15-inch screen, has a standard USB mouse and has a stable internet connection with a web browser installed. These minimal technological requirements for the at-home assessments may facilitate deployment in clinical studies and increase access.

Most participants indicated that they would be willing to complete the full or an abbreviated version of the 4-week study again. Our reliability data indicate that a 2-week data collection period involving 1 week of continuous wearable sensor data collection and twice a week performance of Hevelius for 2 weeks is sufficient.

### Limitations

There are some limitations in our study. There was heterogeneity in the medium-sized cohort, which included 34 individuals with SCA types 1, 2, 3 and 6 and MSA-C, most of whom were recruited from a single site (*n* = 29). Our goal was to identify general motor measures that were applicable across this set of diseases with overlapping phenotypes. Disease-specific models could be trained in the future with larger-scale data collection across multiple sites, which is feasible with these at-home technologies. Given that the primary aim was to establish the relationship between digital motor measures and patient-reported function and ataxia severity, there were a relatively small number of controls included in the study. However, the control sample was sufficient to identify strong and statistically significant differences between ataxia and control participants, including differences between preataxic and control participants, thereby highlighting the sensitivity of these tools. Larger samples of control and preataxic individuals will be needed in future research to characterize the ability of sensor and computer mouse measures and models to sensitively identify disease onset and measure small changes in the earliest stages of disease. Furthermore, longitudinal studies will be necessary to assess sensitivity of these tools for capturing disease progression.

## Conclusion

In summary, we report on two relatively inexpensive, easy-to-use and fully remote quantitative assessment technologies that demonstrate potential for use as motor outcome measures in clinical trials. Continuous ankle sensor-based measures and Hevelius computer mouse task measures correlated strongly with both patient-reported measures of function and ataxia rating scales, had very high test–retest reliability and strongly distinguished between ataxia and control participants, including preataxic and control participants in some cases. The ankle sensor composite measures are based on interpretable SM features that have specific relevance to the ataxia phenotype and more generally to the control of movement. These real-life ankle movement measures correlated more strongly with patient-reported function than task-based clinical ataxia rating scales, further supporting that they capture aspects of movement that are meaningful to patients. The cross-sectional properties with high test–retest reliability and sensitive relationships with disease severity suggest sensitivity for measuring disease progression. Longitudinal data are necessary to evaluate and quantify sensitivity to disease change.

## Supplementary Material

fcad064_Supplementary_Data

## Data Availability

Data included in this study will be shared by request from any qualified investigator.

## Abbreviations

AI =: activity intensity
BARS =: Brief Ataxia Rating Scale
es =: effect size
ICC =: intraclass correlation coefficient
MDS-UPDRS =: Movement Disorder Society-Unified Parkinson Disease Rating Scale
MSA-C =: multiple system atrophy, cerebellar type
PC =: principal component
PCA =: principal component analysis
PROM =: Patient-Reported Outcome Measure
SARA =: Scale for the Assessment and Rating of Ataxia
SCA =: spinocerebellar ataxia
SM =: submovement
