# Supplementary material for: Real-life ankle submovements and computer mouse use reflect patient-reported function in adult ataxias
Source: Brain Commun. 2023 Mar 13;5(2):fcad064. doi: 10.1093/braincomms/fcad064 (PMC10042315; doi:10.1093/braincomms/fcad064)
Supplement: fcad064_Supplementary_Data [file fcad064_supplementary_data.pdf]

Supplementary Material

Supplementary Methods

Equipment & Supplies

Most participants (38/42) used a provided study laptop to complete the Hevelius computer mouse task. Participants were required to use a laptop or desktop computer with at least a 15-inch screen, a standard USB mouse, and a stable internet connection with a web browser installed. A web camera was also required for the Zoom appointment. Participants were instructed to perform the computer tasks at the same location in their home each time and use a table or a desk with enough space to comfortably use the mouse. Participants also optionally received a mobile wifi hotspot and charging cable if they were concerned about their wifi connection signal or were unsure how to connect the laptop to their home wifi network. Participants received an instructional sheet and diagram describing how to set up for the study (Supplementary Fig. 1).

All participants were provided with two GENEactiv wearable sensor devices, one for the dominant wrist and one for the dominant ankle. The device measures tri-axial acceleration at 100 Hz with a MEMS sensor (range: +/-8g; res: 12bit). The study supplies were mailed to the participant's home address. Participants began the wear-period after being guided by a study coordinator on how to properly wear and activate the devices during the initial Zoom video conference call. The devices were set up so that once they were activated, they were unable to be turned off. Participants were asked to wear these devices continuously for one week and were reminded that they were waterproof and should be worn while sleeping. The small and lightweight device could be worn continuously for the week without the need to recharge or download data from the device. The wrist device, worn like a watch with a waterproof rubber band, included an adjustable strap to ensure the device was securely fastened on the wrist. Originally, the cloth ankle band that was worn was fastened with velcro and would occasionally cause skin irritation. Four participants reported discomfort with the original band, so this was replaced with a more comfortable band that was used by participants 14-42. Zero of the last 29 participants experienced discomfort with the new ankle band. The ankle device was placed inside of a fabric band with secure snap buttons so

it could be worn comfortably around the ankle. Of note, the algorithms used in data analysis do not require the sensor to be oriented a specific way, thus the motor measures were tolerant to errors in placement. Although the GENEactiv device was chosen for use in this study, since only triaxial accelerometer data were used, there is potential for the same motor measures to be obtained from other similar devices.

Questionnaires

Study questionnaires listed below were collected and managed using REDCap[1] electronic data capture tools hosted at MGH.

Rand 36 Item Short Form Health Survey: The Rand Short Form 36[2] is a 36-item patient-reported health survey that covers health realms domains of physical functioning, emotional well-being, energy/fatigue, social functioning, pain, and general health.

EQ-5D-5L and EQ-VAS: The EQ-5D-5L[3,4] is a brief, patient-reported health survey that covers the patient's health, mobility, self-care, usual activities, pain/discomfort, and anxiety/depression. The EQ-VAS (Visual Analog Scale) is a patient-reported visual scale that assesses a patient's current health on a scale from 0 (worst imaginable health) to 100 (best imaginable health).

Neuro-QOL Fatigue scale: The Neuro-QOL Fatigue subscale[5] is a brief, 8-item scale used to assess participants' feelings of fatigue over the period of the past 7 days.

PROM-Ataxia scale: The PROM-Ataxia scale is a 70-item survey, consisting of 5 sections that assess physical function (2 sections), activities of daily living (1 section), and mental function (2 sections) in individuals with cerebellar ataxia.[6] The score ranges 0-280, with higher scores indicating more impaired function. These 70 questions were divided into subsections for analysis: motor (28), symptoms (25), emotion (10), cognition (7), arm (15), gait (12), speech (2), swallowing (2), and communication (3) (see Supplementary Table 1).

Study Feedback Survey: This survey was administered to all participants in the study, regardless of completion status. The survey asked the participants their opinions on each of the study task's difficulty level, time commitment, and instruction clarity, the setting and device(s) used to complete each study task, and whether they experienced any problems while completing each task. At the end of the survey, participants selected symptoms that they typically experience (from a list

of common symptoms of SCA and MSA), and were asked to rate if the study tasks were able to capture each symptom (on a scale including 2, Yes/mostly; 1, possibly/unsure; or 0, No/not really). They then had the opportunity to provide comments for the study team.

Supplementary Tables and Figures

Supplementary Table 1. PROM-Ataxia subscores. Motor, symptoms, emotion, and cognition subscores were taken directly from Schmahmann et al.,[6] whereas arm and gait/balance subscores were generated based on the PROM-Ataxia questions listed.

**Subscore**	**Question**
Motor	Labeled as “Physical” in Section 2 of PROM-Ataxia
Symptoms	Labeled as “Physical” in Section 1 of PROM-Ataxia
Emotion	Labeled as “Mental” in Section 1 of PROM-Ataxia
Cognition	Labeled as “Mental” in Section 2 of PROM-Ataxia
Arm score (constructed)	#7: I have trouble controlling movement of my limbs (arms, legs, hands, feet)
	#8 : My hands shake and/or tremor at rest
	#9: My hands/arms shake and/or tremor when doing tasks (such as reaching, carrying, pouring)
	#33: I can perform tasks with my hands (e.g., fine motor; grasp things, play an instrument)
	#34: I have control over the use of my arms
	#35: I can write legibly
	#36: I can type on a keyboard
	#38: I can do household work by myself (e.g., cleaning, laundry, making the bed, lifting, carrying)
	#39: I can do yardwork like gardening, raking leaves, weeding, mowing the lawn
	#40: I can go shopping by myself for groceries, clothes, household items

	#44: I can cook on my own
	#45: I can cut food, handle utensils, and put on jewelry without assistance
	#46: I can dress myself, including tying shoelaces, buttoning my clothes, putting on socks, earrings, watch, belt
	#49: I can brush my teeth without assistance
	#50: I can shave my face or apply makeup without assistance
Gait and balance score (constructed)	#1: I feel unsteady on my feet when standing/walking on flat surfaces.
	#2: I lose my balance walking/hiking on uneven surfaces, including hills and sand/beach
	#3: I feel as though I may lose my balance and fall
	#4: I lose my balance on stairs/ladders/stepstools
	#6: I find myself stumbling and/or falling
	#27: I can walk without assistance (without a cane, walker, personal aid, wheelchair)
	#28: I can catch myself and prevent a fall when I stumble
	#29: I am able to engage in the sport of my choice (e.g., running, horseback riding, bicycling, golf)
	#30: I can bend down and pick something off the floor without help
	#47: I can move about the house without assistance
	#51: I can get in and out of bed without assistance
	#52: I can get on and off the toilet without assistance

Supplementary Table 2. Participant clinical data grouped by diagnosis.

Diagnosis	Score	Range	Mean	Standard Deviation
**SCA1 (n=3)**	BARS total	9.75-13.75	11.5	2.05
	SARA total	11-14.09	12.83	1.62
	UPDRS total	17.5-37	30.23	11.03
	PROM-Ataxia total (week 1)	73-120	95.33	23.59
**SCA2 (n=2)**	BARS total	9.5-17	13.25	5.3
	SARA total	11.75-16.75	14.25	3.54
	UPDRS total	26-29.5	27.75	2.47
	PROM-Ataxia total (week 1)	113-137	125	16.97
**SCA3 (n=20)**	BARS total	0.25-21	10.31	6.24
	SARA total	0.75-25.75	11.43	6.95
	UPDRS total	1-50.48	21.9	14.17
	PROM-Ataxia total (week 1)	21-162	90.85	45.91
**SCA6 (n=3)**	BARS total	2.5-5.5	4.5	1.73
	SARA total	1-5	3	2
	UPDRS total	4-12	7.33	4.16
	PROM-Ataxia total (week 1)	51-67	59.33	8.02
**SCA (all types) (n=28)**	BARS total	0.25-21	10.03	5.78
	SARA total	0.75-25.75	10.87	6.58

	UPDRS total	1-50.48	21.65	13.62
	PROM-Ataxia total (week 1)	21-162	90.39	41.7
**MSA (n=6)**	BARS total	7.25-18.5	14.58	4.13
	SARA total	8.25-20.19	16.39	4.34
	UPDRS total	14.5-56.6	34.11	15.43
	PROM-Ataxia total (week 1)	118-187	147	28.44
**Control (n=8)**	BARS total	0-1.5	0.19	0.53
	SARA total	0-3.5	0.44	1.24
	UPDRS total	0-7	1.25	2.43
	PROM-Ataxia total (week 1)	9-55	25.5	15.11

Supplementary Table 3. Hevelius computer mouse task regression model weights. Each column represents a single disease severity estimation model. The values shown in each column are the weights assigned to each of the task features for that given model. Feature weights of '0.00' or less are omitted for clarity. Model weights differ from those reported in Gajos et al.[7] as the models were retrained since the publication.

Abbreviations: CV – Coefficient of Variation; SD – Standard deviation; SM – Submovement; BARS – Brief Ataxia Rating Scale; UPDRS – Unified Parkinson's Disease Rating Scale; Comp – Comparisons.

	BARS Total Prediction Model	BARS Arm Prediction Model	UPDRS Total Prediction Model	UPDRS Arm Prediction Model	Pairwise Comp Total Prediction Model	Pairwise Comp Arm Prediction Model
Movement Time	2.13	0.12			1.11	0.97
Movement Time (CV)					-0.15	-0.70
Execution Time	0.31	0.02	2.76		0.60	0.56
Execution Time (CV)					-0.27	0.47
Execution Time Without Pauses					-0.70	-0.94
Execution Time Without Pauses (CV)		0.03			0.31	0.35
Verification Time	-0.78	-0.03		0.45	-0.39	-0.19
Verification Time (SD)			0.60	0.20	-0.05	0.13
Number of Pauses	0.20	0.04			-1.05	1.16
Duration of Longest Pause					0.63	-0.80
Max Speed	0.20		-1.50	-0.18	0.18	0.78
Max Speed (CV)					0.17	0.51
Max Acceleration					-0.36	-1.12
Max Acceleration (CV)					-0.64	-0.69
Normalized Jerk				0.51	-0.10	-1.21
Normalized Jerk Without Pauses					0.28	1.56
Click Duration	0.10	0.10	0.40	0.15	-0.10	0.19
Click Duration (SD)			1.27			-0.08
Movement Direction Changes		0.06			0.40	0.60
Orthogonal Direction Changes	0.08				0.24	-0.82
Task Axis Crossings			-3.15		-1.58	-1.61
Max Deviation from Task Axis					-2.05	-0.45
Movement Error			-0.66		-2.32	-4.93
Movement Offset					-0.66	-0.14
Movement Variability				-0.69	4.49	4.91
Distance from Target at end of Main SM	0.12				0.15	-0.06
Target Re-entries		0.01			0.27	0.53
Click Slip	0.39		1.23	0.64	0.41	0.58
Fraction Distance Covered in Main SM	0.29	0.04	1.18	0.08	0.24	0.31
Fraction of Main SM Spent Accelerating			2.63	0.61	0.21	0.19
Number of Submovements			1.84		0.22	0.67
Main Submovement		0.08			-0.13	0.13
Noise to Force Ratio				0.13	0.60	0.42

Supplementary Table 4. Relationships between clinical rating scales and PROM-Ataxia. Levels of significance are marked as follows: p< 0.05(*), p<0.001(**)

	BARS Total	*BARS Gait*	*BARS Dominant Arm*	*SARA Total*	*SARA Gait*	*SARA Dominant Arm*	*UPDRS Total*	*UPDRS Gait*	*PROM Ataxia Total*	*PROM Ataxia Symptom Score*	*PROM Ataxia Motor Score*	*PROM Ataxia Arm Score*	*PROM Ataxia Gait Score*	*PROM Ataxia Cognitive Score*	*PROM Ataxia Emotion Score*
BARS Total	1														
BARS Gait	0.94**	1													
BARS Dominant Arm	0.89**	0.80**	1												
SARA Total	0.97**	0.95**	0.88**	1											
SARA Gait	0.94**	0.99**	0.79**	0.94**	1										
SARA Dominant Arm	0.79**	0.69**	0.94**	0.76**	0.68**	1									
UPDRS Total	0.88**	0.86**	0.80**	0.91**	0.84**	0.68**	1								
UPDRS Gait	0.94**	0.95**	0.86**	0.96**	0.95**	0.76**	0.87**	1							
PROM Ataxia Total	0.75**	0.80**	0.63**	0.76**	0.76**	0.60**	0.70**	0.79**	1						
PROM Ataxia Symptom Score	0.65**	0.75**	0.53**	0.67**	0.71**	0.45**	0.59**	0.73**	0.91**	1					
PROM Ataxia Motor Score	0.80**	0.82**	0.71**	0.82**	0.79**	0.69**	0.73**	0.83**	0.95**	0.78**	1				
PROM Ataxia Arm Score	0.80**	0.83**	0.69**	0.80**	0.80**	0.66**	0.70**	0.82**	0.94**	0.81**	0.97**	1			
PROM Ataxia Gait Score	0.81**	0.86**	0.70**	0.83**	0.83**	0.67**	0.76**	0.86**	0.93**	0.82**	0.96**	0.91**	1		
PROM Ataxia Cognitive Score	0.43*	0.42*	0.28	0.38*	0.39*	0.27	0.39*	0.35*	0.67**	0.47**	0.62**	0.65**	0.53**	1	
PROM Ataxia Emotion Score	0.17	0.26	0.10	0.20	0.20	0.11	0.31	0.20	0.57**	0.48**	0.37**	0.36*	0.39*	0.52*	1

Supplementary Table 5. Test-retest reliability of patient-reported outcomes.

Questionnaire	Subscore	ICC
**PROM-Ataxia**	Total Score	0.95
	Symptom	0.95
	Motor	0.95
	Emotion	0.79
	Cognitive	0.71
**EQ-5D-5L**	Mobility	0.89
	Self-Care	0.62
	Usual Activities	0.82
	Pain/Discomfort	0.40
	Anxiety/Depression	0.75
	VAS Scale	0.79
**Neuro-QOL**	T-score	0.85
	Standard Error	0.82
	Q1 (13)	0.73
	Q2 (11)	0.65
	Q3 (15)	0.63
	Q4 (6)	0.67
	Q5 (7)	0.71

	Q6 (10)	0.82
	Q7 (14)	0.74
	Q8 (2)	0.51

Supplementary Table 6. Properties of wrist sensor features. Relationships with ataxia rating scales and patient-reported function, test-retest reliability, and disease versus control statistics are provided. Key features/models are bolded. Relationships that are not significant are labeled as "n.s.". Note: the p-values reported for the relationships with the ataxia rating scale arm subscore is for the BARS arm subscore as these relationships were stronger than the SARA arm subscore relationships.

Sensor	Feature Name	Statistic	SM Duration Group	Direction of Motion Group	Relationship with SARA (BARS)				Relationship with PROM-Ataxia				Test-retest reliability	Disease vs Control	
					Total		Arm subscore (finger-nose-finger)		Total		Arm subset				
					r	p-val	r	p-val*	r	p-val	r	p-val	ICC	p-val	es
**Wrist (Single Features)**	SM Distance	Mean	Long	PC1	-0.52 (-0.58)	2.0E-03	-0.31 (-0.40)	(3.0E-02)	-0.54	2.0E-03	-0.58	5.0E-04	0.96	n.s.	-
		Mean	Long	PC2	-0.49 (-0.59)	4.0E-03	-0.28 (-0.36)	(5.0E-02)	-0.56	1.0E-03	-0.59	5.0E-04	0.95	n.s.	-
		**Mean**	**Short**	**PC1**	**-0.64 (-0.68)**	**1.0E-03**	**-0.46 (-0.56)**	**(2.0E-02)**	**-0.66**	**6.0E-04**	**-0.68**	**4.0E-04**	**0.82**	**n.s.**	**-**
		**Mean**	**Short**	**PC2**	**-0.61 (0.65)**	**2.0E-03**	**-0.43 (-0.53)**	**(2.0E-02)**	**-0.64**	**5.0E-04**	**-0.65**	**5.0E-04**	**0.93**	**n.s.**	**-**
		SD	Long	PC1	-0.56 (-0.59)	2.0E-03	-0.35 (-0.43)	(2.0E-02)	-0.54	2.0E-03	-0.59	5.0E-04	0.95	n.s.	-
		**SD**	**Long**	**PC2**	**-0.56 (-0.63)**	**2.0E-03**	**-0.36 (-0.42)**	**(2.0E-02)**	**-0.60**	**7.0E-04**	**-0.63**	**5.0E-04**	**0.94**	**n.s.**	**-**
		SD	Short	PC1	-0.54 (-0.57)	2.0E-03	-0.46 (-0.52)	(1.0E-02)	-0.58	8.0E-04	-0.57	6.0E-04	0.72	n.s.	-
		**SD**	**Short**	**PC2**	**-0.57 (-0.60)**	**2.0E-03**	**-0.44 (-0.52)**	**(8.0E-03)**	**-0.58**	**7.0E-04**	**-0.60**	**5.0E-04**	**0.89**	**n.s.**	**-**
	SM Velocity	**Mean**	**Long**	**PC1**	**-0.56 (-0.61)**	**2.0E-03**	**-0.34 (-0.44)**	**(2.0E-02)**	**-0.58**	**7.0E-04**	**-0.61**	**5.0E-04**	**0.97**	**n.s.**	**-**
		**Mean**	**Long**	**PC2**	**-0.54 (-0.62)**	**2.0E-03**	**-0.32 (-0.41)**	**(3.0E-02)**	**-0.59**	**7.0E-04**	**-0.62**	**5.0E-04**	**0.95**	**n.s.**	**-**
		Mean	Short	PC1	-0.53 (-0.54)	3.0E-03	-0.36 (-0.48)	(1.0E-02)	-0.52	3.0E-03	-0.54	2.0E-03	0.56	5.0E-02	0.9
		Mean	Short	PC2	-0.57 (-0.60)	2.0E-03	-0.39 (-0.51)	(8.0E-03)	-0.59	7.0E-04	-0.60	5.0E-04	0.83	n.s.	-
		**SD**	**Long**	**PC1**	**-0.59 (-0.60)**	**2.0E-03**	**-0.38 (-0.46)**	**(2.0E-02)**	**-0.57**	**7.0E-04**	**-0.60**	**5.0E-04**	**0.97**	**n.s.**	**-**
		**SD**	**Long**	**PC2**	**-0.57 (-0.63)**	**2.0E-03**	**-0.39 (-0.44)**	**(2.0E-02)**	**-0.60**	**6.0E-04**	**-0.63**	**4.0E-04**	**0.95**	**n.s.**	**-**
		SD	Short	PC1	-0.43 (-0.45)	2.0E-02	-0.35 (-0.44)	(2.0E-02)	-0.44	2.0E-02	-0.45	8.0E-03	0.62	n.s.	-
		**SD**	**Short**	**PC2**	-0.51 (-0.54)	3.0E-03	-0.39 (-0.50)	(9.0E-03)	-0.52	3.0E-03	-0.54	2.0E-03	0.86	n.s.	-
	SM Acceleration	**Mean**	**Long**	**PC1**	**-0.59 (-0.63)**	**2.0E-03**	**-0.41 (-0.49)**	**(2.0E-02)**	**-0.63**	**4.0E-04**	**-0.63**	**4.0E-04**	**0.97**	**n.s.**	**-**
		**Mean**	**Long**	**PC2**	**-0.56 (-0.62)**	**2.0E-03**	**-0.37 (-0.44)**	**(2.0E-02)**	**-0.64**	**6.0E-04**	**-0.62**	**4.0E-04**	**0.96**	**n.s.**	**-**
		Mean	Short	PC1	-	n.s.	-	n.s.	-0.32		-0.35	5.0E-02	0.30	n.s.	-
		Mean	Short	PC2	-0.43 (-0.47)	2.0E-02	-0.27 (-0.41)	(3.0E-02)	-0.45	9.0E-03	-0.47	6.0E-03	0.68	n.s.	-
		SD	Long	PC1	-0.56 (-0.59)	2.0E-03	-0.40 (-0.48)	(1.0E-02)	-0.60	7.0E-04	-0.59	5.0E-04	0.87	n.s.	-
		SD	Long	PC2	-0.53 (-0.59)	2.0E-03	-0.37 (-0.45)	(2.0E-02)	-0.61	7.0E-04	-0.59	5.0E-04	0.86	n.s.	-
		SD	Short	PC1	-	n.s.	-	n.s.	-0.27		-0.30		0.57	n.s.	-
		SD	Short	PC2	-0.35 (-0.39)	5.0E-02	-	n.s.	-0.37	4.0E-02	-0.39	3.0E-02	0.79	n.s.	-
	AI	Mean	N/A	N/A	-0.63 (-0.64)	1.0E-03	-0.54 (-0.55)	(1.0E-02)	-0.58	7.0E-04	-0.59	5.0E-04	0.83	n.s.	-
		Entropy	N/A	N/A	-0.59 (-0.58)	2.0E-03	-0.51 (-0.52)	(8.0E-03)	-0.57	7.0E-04	-0.52	2.0E-03	0.92	n.s.	-

Supplementary Figure 1. Study setup instruction image (A), sensors as worn during the study (B), and computer mouse task screenshot (C).

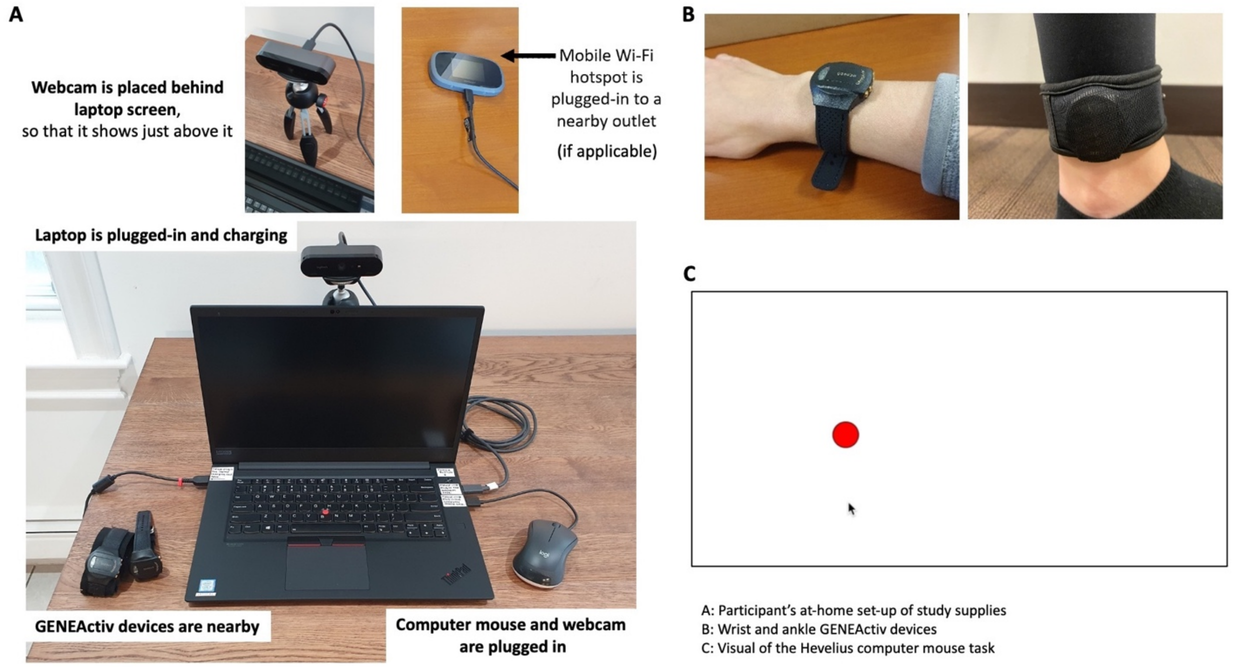

Supplementary Figure 2. Properties of a Hevelius composite model: pairwise comparison regression model. (**A,B**) Relationship of the feature with SARA total score and BARS arm subscore. (**C,D**) Relationship of the feature with PROM-Ataxia total score and arm subscore. (**E**) Test-retest reliability of the feature. (**F**) Disease versus control violin plot.

Abbreviations: *SARA* Scale for the Assessment and Rating of Ataxia; *BARS* Brief Ataxia Rating Scale; *PROM-Ataxia* Patient-Reported Outcome Measure of Ataxia

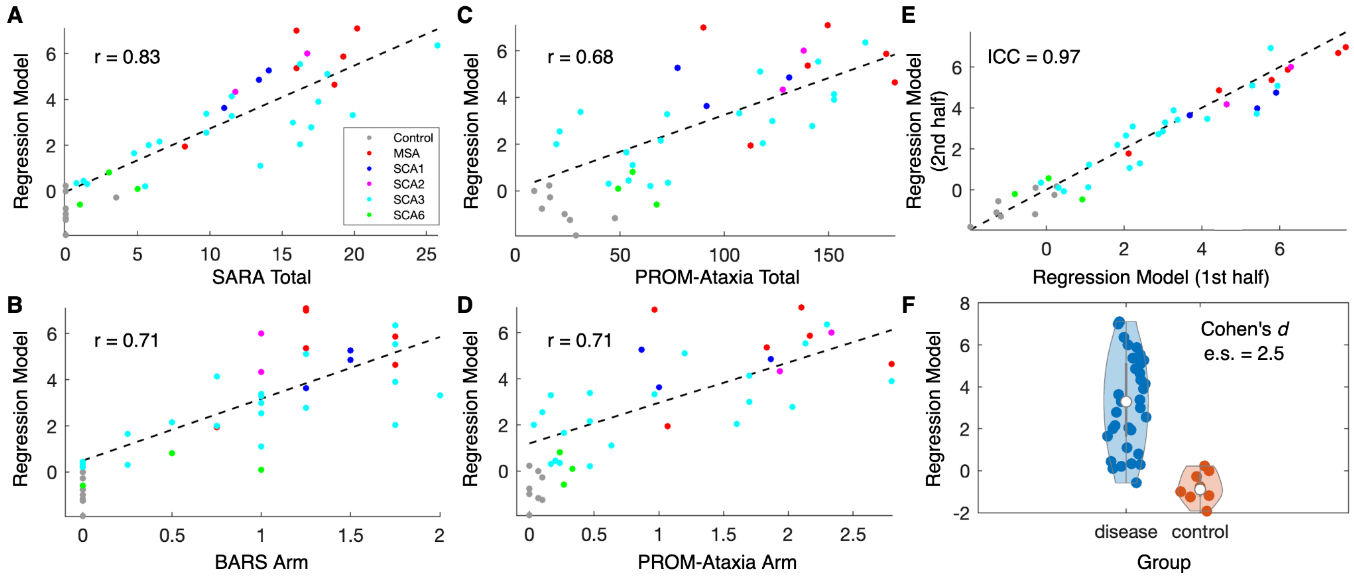

References

1. Harris PA, Taylor R, Thielke R, Payne J, Gonzalez N, Conde JG. Research electronic data capture (REDCap)—A metadata-driven methodology and workflow process for providing translational research informatics support. *Journal of Biomedical Informatics*. 2009;42(2):377-381. doi:10.1016/j.jbi.2008.08.010

2. Hays RD, Hays RD, Sherbourne CD, Mazel RM. *User's Manual for the Medical Outcomes Study (MOS) Core Measures of Health-Related Quality of Life*. RAND Corporation; 1995.

3. Herdman M, Gudex C, Lloyd A, et al. Development and preliminary testing of the new five-level version of EQ-5D (EQ-5D-5L). *Qual Life Res*. 2011;20(10):1727-1736.

4. EuroQol Group. EuroQol--a new facility for the measurement of health-related quality of life. *Health Policy*. 1990;16(3):199-208.

5. Cella D, Hahn EA, Jensen SE, et al. *Patient-Reported Outcomes in Performance Measurement*. RTI Press; 2015.

6. Schmahmann JD, Pierce S, MacMore J, L'Italien GJ. Development and Validation of a Patient-Reported Outcome Measure of Ataxia. *Mov Disord*. 2021;36(10):2367-2377.

7. Gajos KZ, Reinecke K, Donovan M, et al. Computer Mouse Use Captures Ataxia and Parkinsonism, Enabling Accurate Measurement and Detection. *Movement Disorders*. 2020;35(2):354-358. doi:10.1002/mds.27915
